# New insights into the Trans-Saharan gold trade in the Islamic Middle Ages revealed by multi-isotopic (Pb–Fe–Cu) and elemental characterization of Fatimid gold coins

**DOI:** 10.1371/journal.pone.0353759

**Published:** 2026-07-28

**Authors:** Louise de Palaminy, Sandrine Baron, Franck Poitrasson, Robert Kool, Maryse Blet-Lemarquand, François-Xavier Fauvelle

**Affiliations:** 1 Travaux et Recherches Archéologiques sur les Cultures, les Espaces et les Sociétés, TRACES UMR 5608, CNRS – Université Toulouse Jean Jaurès – Ministère de la Culture – EHESS – INRAP, Toulouse, France; 2 Géosciences Environnement Toulouse, GET UMR 5563, CNRS – IRD – Université de Toulouse – CNES, Toulouse, France; 3 Institut des sciences analytiques et de physico-chimie pour l’environnement et les matériaux, IPREM UMR 5254, CNRS, Université de Pau et des Pays de l’Adour, Pau, France; 4 Department of Coins, Israel Antiquities Authority, Jerusalem, Israel; 5 Institut de Recherche sur les ArchéoMATériaux IRAMAT-CEB, UMR 7065, CNRS – Université d’Orléans, Orléans, France; 6 Collège de France, Paris, France; 7 Centre de Recherche Français à Jérusalem UMIFRE 7 UAR 3132, CNRS – MEAE, Jerusalem, Israel; University of Padova: Universita degli Studi di Padova, ITALY

## Abstract

The use of West African gold in medieval times is thought to have enabled the first globalized trade in history: the Trans-Saharan Trade, through the minting of Islamic dinars, which were considered as the equivalent of today’s dollar or euro currencies. Yet, the precise origin of this gold remains debated, as archaeological evidence for such trade is scarce. While textual sources are numerous, they provide only indirect accounts, and the large corpus of surviving dinars has so far yielded limited insight as elemental analyses are not discriminating enough to establish geological provenance. To address this, we combined multi-elemental analysis by LA-ICP-MS with multi-isotope (Pb, Cu, and Fe) data measured by MC-ICP-MS after wet purification chemistry on 11 dinars. This revealed two distinct metal stocks, differentiated by PGE concentrations and Cu isotopes. The Cu and Fe isotope signatures are consistent with supergene and oxidized ores, typical of West African gold mineralization. In contrast, the lead isotopes and calculated model ages of the gold coins do not match the signatures expected for West African mineralization, suggesting that a different, exogenous source is involved in the gold *chaîne opératoire*. These new data then raise two key historical questions: the location of gold processing, whether it occurred North or South of the Sahara, and the hypothetical reuse of older metal stocks.

## Introduction

**Fig 1:**
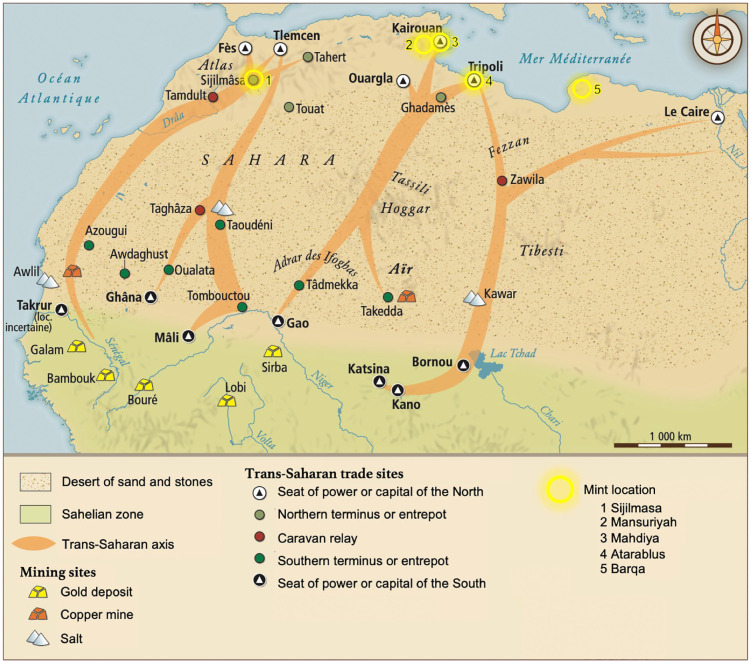
Map of medieval trans-Saharan trade, with cities where coins from the Caesarea treasure were minted. Reprinted from Fauvelle [12] under a CC BY license, with permission from François-Xavier Fauvelle, original copyright CC BY 2018.

The dinar, the Arabic term for gold coinage, played a pivotal role in both everyday transactions and long-distance trade throughout the medieval Islamic world (7th–15th centuries), spanning from al-Andalus (Islamic Spain) to the Indian subcontinent. Across this region the gold dinar, whose weight (up to the end of 11th century) and fineness (up to the 15th century) remained relatively well regulated, facilitated the circulation of commodities, merchants, and monetary, religious and esthetic values across the Old World, i.e., Africa and Eurasia, during the “global” Middle Ages [1–11]. Yet the origins of the gold used to mint these coins remain an open and intriguing question. The Islamic world lacked direct access to most of the major gold sources, especially those of the Balkans and the Caucasus. All known gold mines on the Iberian Peninsula date to Roman times (or the 19th century) presumably due to the considerable resources required to exploit such unprofitable deposits. It is therefore widely accepted that a significant—if not predominant—portion of the gold used in Islamic coinage originated in Sub-Saharan Africa, notably from regions such as West Africa referred to as Sudan in Medieval Arabic sources. This hypothesis is supported by several strands of evidence. Medieval Arabic sources—often indirect and based on the testimonies of Muslim merchants and travelers—frequently mention gold (alongside enslaved persons) as a major commodity exported northward across the Sahara and the Indian Ocean. Numismatic data, including the dates and mint locations inscribed on most Islamic coins, indicate that key minting centers were positioned at the northern termini of trans-Saharan trade routes (**Fig 1**). Comparative ethnographic data also offer glimpses into premodern gold extraction and processing techniques [4,13,14].

Nonetheless, direct material evidence of the trans-Saharan gold trade remains scarce. Archaeological findings from known gold-producing regions in Sub-Saharan Africa are limited [4,15], and the *chaîne opératoire*—the sequence of technical steps transforming raw gold into coin—remains largely undocumented. To date, only a few relevant material traces have been uncovered, notably crucible fragments from the late medieval site of Begho in present-day Ghana [16] and from the Saharan emporium of Tadmekka in present-day Mali [17,18].

A promising alternative line of inquiry lies in the elemental analysis of the metal itself contained in Islamic gold objects [6,17,19–24].

It is generally assumed that gold exploited during the medieval period was primarily alluvial, as suggested by medieval Arabic sources stating that gold was collected “like carrots” in Sud-Saharan Africa [4]. Surface-accessible supergene gold would indeed have been easier to extract and process than gold from quartz veins or paleo-placers. However, evidence of medieval extraction from primary deposits exists, for instance, in Egypt’s Eastern Desert [25], and has also been proposed for West Africa [7].

The present-day West African Craton (WAC), particularly its southern part, is exceptionally rich in gold resources [26–28]. Gold occurs primarily in orogenic and placer (i.e., alluvial) deposits, the latter being the most economically significant in pre-industrial times. Other deposit types, including lateritic, porphyry, iron oxide-copper-gold (IOCG), and intrusion-related mineralizations, are less common. The major phase of gold mineralization in the WAC occurred during the Paleoproterozoic era. The principal structural event responsible was the Eburnean orogeny (ca. 2.2–2.0 Ga), which formed the gold-bearing Birimian greenstone belts in the southern WAC. Paleoplacer deposits also formed during this period.

Naturally occurring gold in primary ores usually contains significant amounts of silver (up to 40–50%), minor amounts of copper (<1%), and only trace levels of lead and iron [29]. In tropical environments such as West Africa, supergene weathering alters these compositions, producing high-purity gold through silver depletion or selective reprecipitation of gold [30–33]. For instance, hypogene gold typically averages 90% Au and 10% Ag, while supergene gold reaches ~96% Au and 4% Ag, with Cu ranging from to 0.01–0.1% [30]. This could explain why archaeological finds like the prills recovered from suspected gold molds from Tadmekka (Mali), analyzed at 98.5% Au, 1.2% Ag and 0.2% Cu, can be attributed as naturally pure supergene gold rather than the product of deliberate refining, consistent with the crucibles used only to remove coarse impurities [17,18,34].

Broader datasets from Guinea, Sierra Leone, Mauritania, Mali and Côte d’Ivoire confirm these trends: most nuggets contain <8% Ag and <0.4% Cu [29,35], with trace element contents generally depleted in PGE such as Pt and Pd [6,20,21,23]. Such compositions are compatible with Messier’s [19] early hypothesis that Almoravid dinars were minted from largely unrefined West African gold (~92% Au, 8% impurities). Subsequent studies proposed that very low Pt (1–3 µg/g) and somewhat elevated Sb in coins might represent diagnostic markers for this origin [6,21].

However, despite recurring patterns of high fineness and low trace element contents, West African gold does not display a unique geochemical “fingerprint”. Elemental composition can characterize regional metal stocks but cannot definitively identify ore provenance for individual artifacts [36]. If Pt and Pd remain among the most promising discriminants [37–39], the natural heterogeneity of alluvial deposits [29,40,41], combined with the potential for loss or addition of elements during the chaîne opératoire (be it intentional or unintentional), means that it is extremely likely that elemental signatures were modified along the sequence of transformation of gold.

By contrast, stable, and especially radiogenic isotopic ratios are more robust indicators of provenance. Governed by more specific physical-chemical partitioning processes relative to elemental concentration [42], isotopic signatures preserve geological information more reliably relative to elemental concentrations and offer a more powerful tool for tracing the origin of ancient gold [24,41,43–60]. Even though the stable isotope approach to trace archaeological metals was sometimes criticized on the basis of the finding of large local isotopic heterogeneity of some ore deposits [61,62], it appears that, besides possible analytical issues, these previous studies largely ignored the geological and particularly archaeological context of the ore collection and treatment by the ancient miners before metal object production [63,64].

Hence, the present study seeks to address this new approach by applying multi-isotopic (Pb, Fe, Cu) with multi-elemental analyses to Fatimid gold coins, in order to shed new light on the trans-Saharan gold trade and its associated *chaîne opératoire* from the mine to the mint.

### Corpus

The number of Early Islamic single gold coins and hoards discovered in excavations over the past decades has grown exponentially, particular those minted during the Fatimid period (10th-11th c.) [65]. As a result, numismatic research has made impressive advances with publication of a detailed corpus of these coins [66,67]. In 2015, a hoard of 2,668 gold coins of this period, one of the largest hoard of its kind ever discovered in the Eastern Mediterranean (weighing 7.5 kg), was found in a shipwreck in the ancient harbor of Caesarea, Israel [68]. Now housed in the Coin Department of the Israel Antiquities Authority in Jerusalem, this assemblage offers a rare and valuable case study for gold provenance research. It presents two major advantages: it is both “sourced” and “sealed.” First, its archaeological context is securely known—unlike coins in most numismatic collections, which mostly derive from undocumented donations. Second, having remained underwater for nearly a millennium, the hoard has not been subject to post-depositional processes such as reminting, recycling, or forgery, nor chemical cleaning, preserving the original metallurgical composition of the coins.

The Caesarea hoard consists of dinars (c. 4g) and quarter dinars (c.1g) minted over a span of approximately 220 years, from the reign of the Aghlabid emir Ziyadat Allah I (816–838) to that of the Fatimid caliph al-Zahir (1021–1035), with a majority issued under the Fatimid dynasty (10th–11th centuries). The Fatimid Caliphate spanned the Maghreb, Sicily, the Levant, and the Arabian Peninsula, with capitals successively located in Raqqada (909–918) and Mahdiya (918–969) in Tunisia, and later in al-Qahirah/Cairo from 969 onwards in Egypt. Nearly 90% of the hoard’s coins were struck at four main mints: Mansuriyah (29%) in Tunisia, Misr/Fustat (27%) in Egypt, Siqilliyah (17%) in Sicily, and Mahdiya (15%) in Tunisia **–** predominantly under the reigns of two successive Fatimid caliphs, al-Hakim (reign 996–1021) and al-Zahir (r. 1021–1036) (**Fig 1**) [66,69]. Smaller subsets were minted across Morocco, Libya, the Red Sea region, Syria, and Yemen. The hoard is exceptional in its size, wide geographical spread, and relatively narrow chronological window.

To investigate the provenance of the gold used in these coins, we sought to compare their chemical signatures across different mints while minimizing temporal variability. To accomplish this, eleven coins from the Caesarea hoard were selected for novel isotopic exploration. (**Table 1**, **Fig 1**). Except for two quarter dinars, all date to the reign of al-Hakim, allowing for a tight chronological control and reducing the likelihood of major shifts in gold supply over time. To avoid coins potentially made from mixed gold stocks, we prioritized specimens from smaller North African mints, particularly those located along the termini of major trans-Saharan trade routes—where gold from West Africa was more likely to have circulated—rather than from central hubs like Fustat, which likely drew from multiple gold sources. The selected coins include specimens from Mansuriyah (coins 006, 005, 859, and 915) and Mahdiya (coins 236 and 010) in Tunisia, which represent the central Saharan trade routes; Barqa (coin 218) and Atarablus (coins 566 and 792) in Libya, linked to the eastern Saharan routes; and two quarter dinars from Sijilmasa (coins 205 and 206) in Morocco, related to the western Saharan routes. Albeit not minted under the same Caliph (al-Hakim did not mint coins at Sijilmasa) but by al-Mahdi (r. 910–934), the latter two specimens were included due to the geographic importance of their minting location. Sijilmasa represents the southernmost Islamic mint known to this day, and is thus the closest to the West African goldfields.

**Table 1:** Corpus of the gold coins.

Sample ID	Accession number	Mint	Present-day country	Type of coin	Mint date	Sovereign
**566**	151566	Atarablus	Libya	Dinar	1009-1010	Al-Hakim
**792**	150792	Atarablus	Libya	Dinar	1020-1021	Al-Hakim
**218**	150218	Barqa	Libya	Dinar	1016-1017	Al-Hakim
**236**	150236	Mahdiya	Tunisia	Dinar	1013-1014	Al-Hakim
**010**	151010	Mahdiya	Tunisia	Dinar	1016-1017	Al-Hakim
**006**	152006	Mansuriyah	Tunisia	Quarter dinar	1009-1021	Al-Hakim
**005**	152005	Mansuriyah	Tunisia	Quarter dinar	1009-1021	Al-Hakim
**859**	150859	Mansuriyah	Tunisia	Dinar	1018-1019	Al-Hakim
**915**	150915	Mansuriyah	Tunisia	Dinar	1010-1011	Al-Hakim
**205**	152205	Sijilmasa	Morocco	Quarter dinar	910-934	Al-Mahdi
**206**	152206	Sijilmasa	Morocco	Quarter dinar	910-934	Al-Mahdi

## Results

All data are provided in S1 Dataset. In this section, we describe the multi-elemental and isotopic analyses. How these data sets correlate with each other will be discussed in the subsequent section.

### The Fatimid gold coins elemental signature

**Fig 2:**
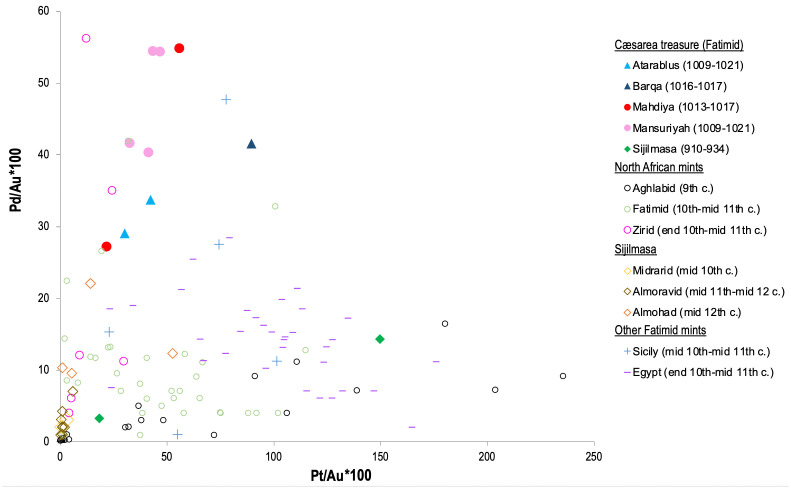
Binary diagram Pd/Au*100 vs. Pt/Au*100 for Caesarea dinars and comparative data from Gondonneau and Guerra [21] with dinars issued in North Africa and especially in Sijilmasa from Aghlabids (9thc.), Fatimid (10th-mid 11th c.), Zirid (end 10th-mid 11th c.), Midrarid (mid 10th c.), Almoravid (mid 11th-mid 12th c.) and Almohad (mid 12th c.) dynasties, and issued in Sicily and Egypt during the Fatimid dynasty (mid 10th-mid 11th c.). Pt and Pd (µg/g) are standardised against Au (%) since these elements are derived from gold, and the ratios have been multiplied by a factor of 100 to ensure comparability.

Elemental results measured by LA-ICP-MS are reported in Table S1 in S1 Dataset. These include major (Au, Ag), minor (Cu) and trace elements (Fe, Pb, Co, Ni, Zn, As, Sn, Sb, Te, Pd, Pt, Bi). The dinars contain 89.7–98.5% Au, 1.0–9.5% Ag and 0.24–0.56% Cu. Coins 010 and 006 are noticeable by their lower value in Au (89.7% and 89.9% respectively) and their higher value in Ag (9.5% and 9.3% respectively). Coins 205 and 206 from Sijilmasa, and 218 from Barqa, are the finest, with 98.5%, 98.3% and 97.8% Au respectively. For minor and trace elements, greater variation was observed. Iron contents of the coins ranges from 618 µg/g to 2056 µg/g, and Pb content ranges from 16 µg/g to 211 µg/g. Cobalt, Ni, As, Sb and Bi were counted in a few µg/g, and Zn and Sn contents range from dozens to a few hundred µg/g. The platinum group elements (PGE) Pt and Pd were counted in a few dozen µg/g with an exception of coin 206, minted in Sijilmasa, which had the highest observed Pt content at 147 µg/g (**Fig 2**).

### The Fatimid gold coins Pb, Fe and Cu isotopic signatures

#### Lead isotopes.

Three lead isotopes are radiogenic in origin (^208^Pb, ^207^Pb, ^206^Pb), resulting from the radioactive decay of ^232^Th, ^235^U and ^238^U, respectively, and one is stable (^204^Pb). They have been widely used since the 1980s to determine the provenance of archaeological artefacts [70] and used occasionally for ancient gold provenance research [24,41,46–55,57,58,60]. Lead isotopes can be used to calculate three independent parameters: the geological age of the tectonic province (model age) that characterizes the igneous reservoir in which the ores were formed, and the U/Pb (µ) and Th/U (κ) ratios that characterizes the metal source [71]. Furthermore, they are not known to show mass dependent partitioning through any processes in the metal *chaîne opératoire* [72]. Unlike the stable isotopes of Cu or Fe, Pb isotopes do not fractionate due to high or especially low temperatures and redox processes [72].

**Fig 3:**
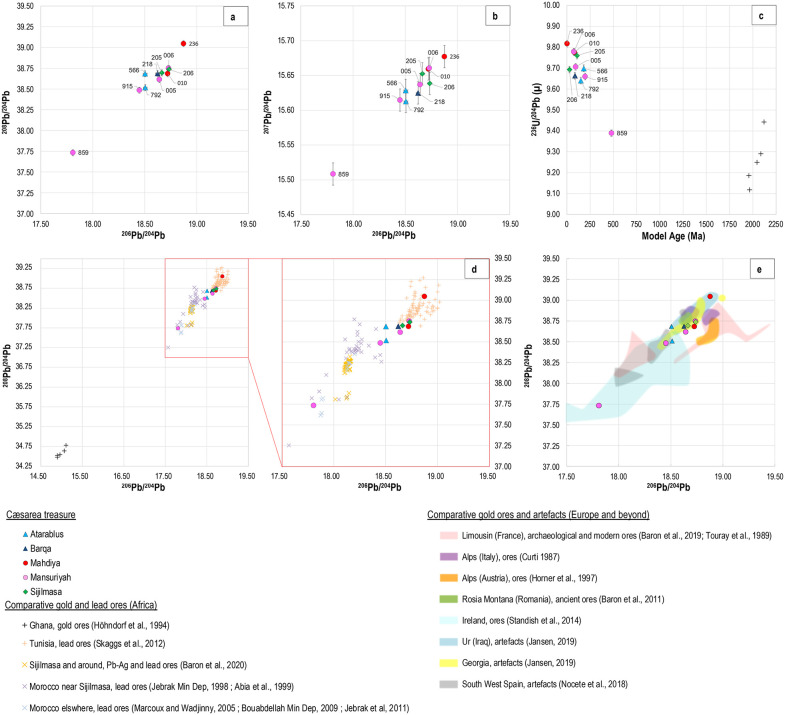
Lead isotopic results of dinars: (a) ^208^Pb/^204^Pb vs. ^207^Pb/^204^Pb and (b) ^208^Pb/^204^Pb vs. ^206^Pb/^204^Pb with the total external uncertainty (2SD), measured against SRM 981 international standard. Triangles stand for mints in Libya, circles for mints in Tunisia and diamonds for mints in Morocco. (c) Binary diagram for inter-comparison between µ and model ages, from Pb isotopes data from Caesarea dinars and Ghana gold ores [73]. The model ages calculated from their data (with Albarède and Juteau [74] model age calculator; recalculation from Stacey and Kramers [75]) are consistent with the ages measured on the host rocks by U-Pb on zircons. The comparison between Pb isotopic data of gold coins and gold ores shows a great difference between model ages and µ. (d) Lead isotopic results for Caesarea dinars compared to North African lead-bearing ores, and gold ores from Ghana: ^208^Pb/^204^Pb vs. ^206^Pb/^204^Pb [73,76–81]. (e) Lead isotopic results for Caesarea dinars compared to available European gold ores (Massif Central in France, Romania, Alps) and artefact from Spain, Mesopotamia and Georgia: ^208^Pb/^204^Pb vs. ^206^Pb/^204^Pb [48,49,55,58,82–84].

The Pb isotopic results are reported in Table S2 in S1 Dataset and **Fig 3**. Ratios for ^208^Pb/^204^Pb fall within the range of 38.5 to 38.8, while the ratios for ^206^Pb/^204^Pb fall between 18.4 to 18.8 except two outliers: coin 859 with less radiogenic value and coin 236 with higher value. These two coins differ from others from the same mints, but their elemental concentrations do not stand out from the rest of the corpus. The two coins from Atarablus share identical ^206^Pb/^204^Pb ratios. All calculated model ages are between 1 and 197 Ma (Cenozoic and Mesozoic) with one exception (coin 859) at ~ 480 Ma (Paleozoic) (Table S3 in S1 Dataset, Fig 3c). Coin 859 stands out with a much lower µ (9.39) than all the other coins (from 9.64 to 9.82) while coin 236 has the highest value. This low µ value would explain the oldest model age for 859, compared to the other coins. Coin 566 stands out (3.98) with a κ higher than the other coins (from 3.85 to 3.94) while coin 236 has this second highest value.

#### Copper and Iron isotopes

Stable isotopes of copper (expressed as δ^65^Cu) and iron (expressed as δ^57^Fe) display signatures associated with types of mineralization (high temperature, hydrothermal or sedimentary ores; primary or secondary ore minerals, e.g., sulphides, oxides, carbonates) according to their fractionation range relative to the continental crust [24,85–89]. These stable isotopic signatures have been used in archaeometry studies to provide evidence for the type(s) of ore mined and/or to indicate geodynamic terranes (e.g., [63,86,87]). Copper isotopes have been used for gold provenance in pionneering studies [24,45,49,57,58,60]. In contrast, Fe isotopes have only been recently used to study archaeological gold [24].

**Fig 4:**
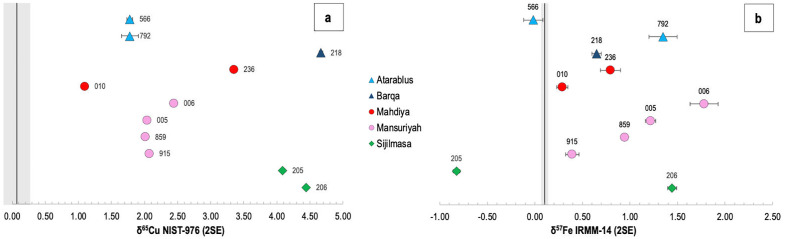
Stable isotopes results. **(a)** Copper isotopic results for dinars (δ^65^Cu, ‰). The dark line represents the Bulk Silicate Earth (BSE) (δ^65^Cu = 0.07) and the shaded area its uncertainty (± 0.10‰ 2SD) [90]. **(b)** Stable Isotopes of Iron from dinars (δ^57^Fe, ‰). The dark line represents the mean mafic Earth isotopic composition (δ^57^Fe = 0.103) and the shaded area its uncertainty(± 0.032‰ 2SE) [91].

Copper isotopic results for our coins range on a broad span—from 1.10 ‰ to 4.67 ‰ (δ^65^Cu) (Table S4 in S1 Dataset, Fig 4a [90]). Copper isotopes appear relatively consistent within each mint, except for Mahdiya. Coins 566 and 792 from Atarablus both show values around 1.78‰. The four coins from Mansuriyah (006, 005, 859, 915), slightly above 2‰, and the two from Sijilmasa (205, 206), slightly above 4‰, each respectively fall within a 0.4‰ range. In contrast, Mahdiya coins 010 and 236 display distinctly different signatures. Note that the isotopic signatures are not homogeneous for the Mahdiya and Mansuriyah workshops, which are close geographically.

Iron isotope compositions (Table S5 in S1 Dataset, Fig 4b) relative to igneous signatures (mean mafic Earth isotopic composition of 0.1‰ in δ^57^Fe), showing a broad distribution range of the coins (> 2.5 ‰) from −0.82 ‰ to 1.78 ‰ (δ^57^Fe). But in contrast to most δ^65^Cu isotopic signatures, this wide range is also visible within each mint. For instance, the Mansuriyah mint represented by four coins (915, 859, 005, 006) has a range of 1.4 ‰, coins from Atarablus mint (566, 792) are separated by 1.4 ‰ and the two coins from Sijilmasa mint (205, 206) are 2.3 ‰ apart.

## Discussion

The origin of the elements in coins is uncertain because they may derive from: (i) the original gold ore itself (ii) additions, for example lead added in the smelting process to separate precious metals (gold and silver) from primary gold ore (iii) debasement by adding silver (silver often comes from silver-lead ore, and thus contains lead) or copper, (iv) purification of gold, via impurities in the added products (e.g., salts, clays) of the cementation process (to separate silver from gold), (v) from any unintentional addition (i.e., contamination) occurring at any moment of the *chaîne opératoire*. It should be noted that these additions, whether voluntary or not, are not always systematic, but they should be evaluated.

Clarifying the possible impacts of the different steps of the *chaîne opératoire* on coins chemical composition is crucial for properly interpreting elemental and Cu, Fe, and Pb isotopic data and, ultimately, to assessing the isotopic signature in terms of the origin of the ores used to produce each coin.

### Interpretation of the Fatimid dinar elemental signature: hypotheses on the *chaîne opératoire* and metal stocks identification

Correlation matrix on elemental composition (details in S1 Fig in S1 Supporting Text) raises issues on the *chaîne opératoire* complexity. Gold is anticorrelated with Cu and Ag, which could reflect their natural occurrence as minor components. Lead content is very low and only weakly correlated with Ag, ruling out intentional silver additions (See details in S1 Supporting Text)—as Ag is produced from lead-bearing ore [35]. With this latter information in mind, the strong correlation of Cu, Ag, and Fe as well as the mildly positive correlation between the trace elements Bi, Zn, As, Sb, and Sn suggests their co-removal during the *chaîne opératoire*, such as by melting, refining or cementation for instance [38,59]. Potential hypotheses are proposed regarding the *chaîne opératoire*, but it should be noted that the coins do not originate from the same workshops or periods (Table 1).

Due to their refractory nature, Pd and Pt were commonly used in previous gold studies (e.g., [6,21,22,38,92–94]) to distinguish metal stocks. In the Pd and Pt (normalized to Au) diagram for the Caesarea dinars (**Fig 2**), two distinct trends emerge, indicating at least two gold metal stocks: one linked to Sijilmasa with low Pd (<15 µg/g) and the other representing various workshops from the early 11th c. with higher Pd (>25 µg/g), with the sole coin from Barqa outlying. The composition of the two Sijilmasa coins, 205 and 206 (early 10th c.) matches with 9th-century Aghlabid coins from North Africa, sharing similar Pt and Pd, as well as Au, Ag, Cu, Sn and Sb contents [21]. These differ from earlier North African coinages, such as Byzantine and Arab-Byzantine coins issued from Carthage (216–444 µg/g Pt, median 338 µg/g, or Pt/Au*100 > 220) [22]. Later Sijilmasa coins (mid-10th to 12th c.) show lower Pt levels (1–20 µg/g Pt, median 3 µg/g, or Pt/Au*100 ~ 3) [21]. Compared with Fatimid coins analyzed by Gondonneau and Guerra [21], the overlap with Caesarea coins is limited, though similar Pt and Pd signatures and Sn and Sb appear in a Fatimid coin from North Africa and Sicily.

Despite the complexity of the gold *chaîne opératoire* and our limited corpus, the method already shows broad trends, and its application to larger and more homogeneous datasets, for instance coins from a single workshop, will yield more robust and historically relevant insights.

### Interpretation of the Fatimid dinar isotopic signature in relation with the *chaîne opératoire*

This section focuses on the isotopic results obtained for Cu, Fe and Pb, and how they provide complementary information on metal stocks, the type of resource, and dinars’ provenance.

#### Copper stable isotopes.

Copper isotope signatures (δ^65^Cu) group coins by individual mint, except for Mahdiya (**Fig 4a**). This suggests two possible explanations: each mint used gold from a distinct source, or each applied a specific gold-processing method affecting the Cu isotopic signature the same way.

If source-related, the heavy δ^65^Cu values (>0.3‰) of dinars point to copper from oxidized or carbonated ores, unlike supergene sulphides (<−0.4‰) or primary sulphides (−0.4 to 0.3‰) [86,95]. In quartz veins or paleoplacers, copper oxides may form co-genetically with gold. In alluvial contexts, i.e., in secondary gold ore types, copper oxides could have been subsequently reduced with gold nuggets and represent a mixture of sources from the drainage basin. Such heavy signatures reflect low-temperature fractionation in supergene, i.e., near-surface processes [95,96]. If copper was cogenetic with gold, it would thus suggest an oxidative or carbonate-type deposit. The Kédougou-Kénieba Inlier (KKI) (2080−2060 Ma) in Eastern Senegal, including the Faleme deposit [97], would match both geologically [27,98] and historically with Islamic sources describing the Bambuk and Bure goldfields, situated between the Upper Senegal, Faleme, and Niger rivers.

If the second hypothesis, related to the chaîne opératoire, is considered, two scenarios emerge.

First, copper could have been added intentionally—through alloying to modify gold properties (but Cu contents are too low to make this option plausible) or through cementation [97,99]—or unintentionally (e.g., via crucibles or workshop dust). The occurrence of several thousand µg/g Cu in gold raises questions, as such levels are unlikely to result from ambient dust, in contrast to Fe. Contamination may reflect proximity to copper metallurgy. Even though surviving goldsmith tools are made of iron, the Islamic tradition of working brass and copper alloys [100] could points to the possibility of combined workshops. The δ^65^Cu signature would reflect the isotopic composition of the added copper, thus from an oxidized/carbonated ore.

Second, isotopic fractionation may have occurred during a process in the *chaîne opératoire*. Berger et al. [59] showed that a thorough cementation, which removes >98% of copper, can shift δ^65^Cu significantly: removing 98.4% of Cu results in a + 5.32‰ shift and 99.5% removal gives +11.28‰. The purest coins (205, 206, and 218), i.e., those with the highest Au content, also exhibit the highest δ^65^Cu values and therefore seem to support this hypothesis. However, when calculating the original copper content in the gold for these coins, the estimated values range from 15% to 26% Cu, which significantly exceeds typical levels found in natural gold and is uncommon even in jewellery alloys. Thus, such heavy signatures are unlikely to result from cementation process.

**Fig 5:**
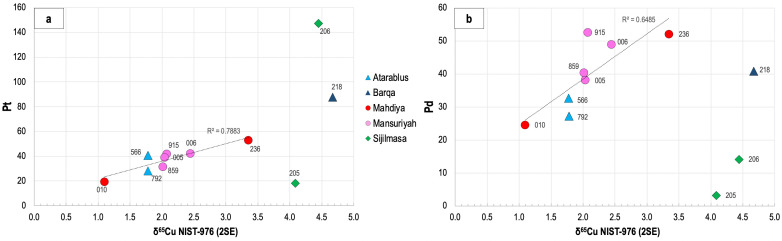
Binary diagrams with (a) Pt (µg/g) vs. δ^65^Cu (‰) and (b) Pd (µg/g) vs. δ^65^Cu (‰) for Caesarea dinars.

Comparing Pt and Pd concentrations with δ^65^Cu values (**Fig 5**), most data points plot along a correlation line (R^2^ = 0.78 and p value = 0.003 for Pt vs ^65^Cu and R^2^ = 0.65 and p value = 0.015 for Pd vs ^65^Cu), except for the two Sijilmasa dinars (205 and 206) and the Barqa dinar (218). Since copper isotopes are not expected to fractionate significantly at the high temperatures involved in metallurgical processes, they are more likely to reflect the isotopic signature of the source ore. Platinum has a very refractory nature. Its content is therefore linked to the gold source and is, with Pd, Pt provides the best elemental tracer for gold [38]. Through this correlation (Fig 5a), it is plausible that both copper and Pt are linked to the source material, bringing together the copper isotope signature and Pt as a workshop signature. The fact that two of the three coins with the highest gold content (205 and 206) deviate from this trend suggests for them a different origin or metallurgical history. This would imply the use of distinct metal stocks for the westernmost mint of Sijilmasa (**Fig 1**), as previously discussed (**Fig 2**), with copper isotopes therefore sharpening the interpretation.

Little is known about other processes, ore extraction or cupellation [97], and therefore in terms of copper isotope effects. From what is known, the heavy δ^65^Cu signatures likely reflect supergene oxidized/carbonated ore, and the preferred hypothesis is that it comes from the gold ore itself with respective supplies to each workshop.

#### Iron stable isotopes.

Currently, the absence of Fe isotopic data on gold materials limits our interpretation. δ⁵⁷Fe values, unlike δ^65^Cu, do not cluster by workshop (**Fig 4b**).

If source-related, the positive and widely dispersed δ⁵⁷Fe values (−0.02 ± 0.10‰ to 1.78 ± 0.15‰ with one at −0.82‰ ± 0.04‰) could reflect the presence of ubiquitous iron-bearing minerals associated with alluvial gold and the minerals involved are more likely of marine and supergene ores relative to igneous origin, such as Banded Iron Formations (BIF) abounding in West Africa [63,87]. This heterogeneity may thus be linked to the variable composition of such minerals. δ⁵⁷Fe can also vary widely within a single deposit (e.g., [101]), making the range more informative than specific absolute delta values.

Alternatively, it could be hypothesized that the Fe signature results from workshop-related contamination, such as from crucibles, tools, or dust—more likely than in the case of copper, given iron omnipresence, higher abundance at the surface of the Earth and its lower concentration in the gold coin studied—or from cementation process [99]. Iron isotope fractionation has not yet been investigated within the gold *chaîne opératoire*; However, Milot et al.[87] reported no evidence of such fractionation in iron metallurgy. Moreover, this contamination effect from workshop dust is unlikely to have resulted into such a strong Fe isotope heterogeneity at the mint scale according to our results (Fig. 4b). This is because contamination from a major element like iron is more likely to represent mixed sources and it should have therefore brought averaged δ⁵⁷Fe signatures close to the continental crust Fe isotope composition [80] which is not the case here.

#### Lead isotopic system.

Mineralisation of gold in the West African Craton took place around 2.0 Ga during the Eburnean orogeny, thus corresponding to ^206^Pb/^204^Pb ratio ~ 15.00 [27,97]. However, the lead isotope data of gold coins are much more radiogenic, > 18.40 (except for 859 gold coin with a ^206^Pb/^204^Pb ratio of 17.81) (Fig 3a-b) highlighting a younger geological age of gold ore sources (Fig 3c). Therefore, these isotopic signatures cannot come from Western African deposits. Eastern Egyptian gold deposits, mineralized during the Late Neoproterozoic c. 600 Ma [102], are also inconsistent because they are too old as well.

The model ages of the coins (all < 200 Ma, except one), along with their µ (~9.72) and κ (~3.90) parameters, align with Mediterranean-type signatures [103]. When plotted in ^208^Pb/^204^Pb vs. ^206^Pb/^204^Pb and ^207^Pb/^204^Pb vs. ^206^Pb/^204^Pb diagrams (**Fig 3d**, [73,76–81]**)**, the data overlap with Tunisian and Moroccan Pb-Ag ores [81], as do the three calculated isotopic parameters. This is a particularly interesting point, given that the coins were minted in North Africa. It should also be noted that the two coins from Sijilmasa do not match Pb-Ag ores from Sijilmasa and its vicinity.

The elemental data do not indicate intentional silver (and thus lead) addition for debasement, as Ag and Pb are not correlated and Pb is too low. Alternatively, lead may have been used in refining impure gold, as mentioned in Islamic sources [104,105], but again, the lack of Pb-Ag correlation argues against cupellation. However, in the case of the cementation experiments carried out by Berger et al.[59], authors were able to report Pb contamination, likely from crucibles or the cement mixture itself, which challenges the reliability of Pb isotopes for provenance studies. As the hypothesis of cementation may be dismissed, on the basis of Cu content and copper isotopes, it may be that the contamination took place at the very beginning of the *chaîne opératoire*. Unintentional Pb contamination via crucibles or heavy minerals associated with gold panning is a credible possibility [106]: a ~ 0.3% contribution of radiogenic exogenous lead can significantly alter isotopic signatures due to very low Pb content in coins (tens to hundreds of µg/g). We thus conclude that local lead from North Sahara was introduced, unintentionally, during the *chaîne opératoire*, thereby hiding the original West African gold signature.

But Pb might also have been unintentionally added earlier in the process, especially for processing gold-rich ores like quartz blocks or paleoplacer conglomerates transported north of the Sahara, as suggested by Fauvelle [7]. In such cases, Pb from geologically distinct sources from the gold could act as a precious metal collector, as seen in Roman practices [48]. This implies ore reduction occurred north of the Sahara, and not south.

Another hypothesis, controversial among historians, is that these dinars were not made from West African gold. In a previous study, this hypothesis has already been raised for dinars from an earlier historical period: Early Islamic coins minted in North Africa might have initially been produced by recycling Roman-Byzantine gold, thus using European-type gold [21,22] and from 750 AD onwards, a shift in gold supply likely occurred, presumably from West Africa (but without any isotopic measurements, it is impossible to recover a geodynamical origin). It has also been demonstrated—based on multi-elemental analyses of jewellery discovered in *tumulus* from Senegal (12th–13th centuries)—that alloyed gold (and silver) was imported from Europe and/or North Africa to West Africa (Magnavita and Mertz-Krauz, 2019), thus pointing to a much more complex trade network. Recycling from older gold stocks cannot be fully excluded, making comparison with European gold ores still relevant. Albeit less likely, the use of northern Mediterranean gold is worth considering. Available Pb isotope data from this region include both ancient and modern gold ores and artefacts—not just Pb ores. These comparisons may, however, be somewhat anachronistic. Comparison between this European data and the Caesarea dinars shows some quite good overlap (**Fig 3e**) [48,49,55,58,82–84]. This underscores the general need for more comprehensive Pb isotopic datasets, including model ages and µ and κ parameters, for gold ores to enable accurate provenance assessments. Lead isotopic signatures can appear similar between some ores and objects while being historically incompatible, highlighting the importance of contextualized archaeological interpretation [107].

Examined in the light of Cu and Fe results, and if considering that these isotopes can trace the gold ores, then they would point to supergene/paleoplacer ores rather than primary gold ores from quartz veins. In other words, this point to gold from very old cratons (West Africa) rather than from more recent geodynamic settings (Southern Europe).

## Conclusions and outlook

Our stable Cu and Fe isotope findings militate for minimal gold metallurgical processing during the production of the dinars. In our previous Cu-Fe-Pb pilot study of gold coins of various ages, it was found that the dinar studied (Ayyubid, 1199–1218) was isotopically much heavier than European gold coins from the 13th, 17th and 19th c. We interpreted this as reflecting the use of ores derived from high-temperature ore-forming processes in the production of more recent gold coins, along with their higher level of metallurgical processing and possibly subsequent mixing of various golds [24]. As a result, more recent European gold coins, especially from 19th c., displayed rather homogeneous Cu and Fe isotope composition, close to the average continental crust, in contrast to the medieval dinar studied in de Palaminy et al.[24] and the additional ones from the present study (Fig 4). Interestingly, the Pb isotopic signature for the dinar from that study yielded a similar model age to those from the present work despite a gap of more than 200 years between the dinar of our pilot study and the corpus of gold coins of this present study. Among the three isotope tracers studied, Pb is the most likely to have been contaminated in the local workshop given its very low concentration in the coins studied relative to Cu and even Fe. It typically displays less than ten times iron concentrations, whereas copper is only of a factor of two to four higher than iron (Table S1 in S1 Dataset). This lead contamination is attested by its isotopic signature and model ages, akin to the local North African lead (Fig 3c-d).

In contrast, it is unlikely that the isotopically heterogeneous iron, at all scales, and the homogeneous copper, at the mint scale, can also be explained by workshop contamination. The first reason is because of the correlation of copper isotopes results and PGE contents (particularly Pt), related to metal stocks due to their refractory nature (Fig 5a). Secondly and most importantly, this is because iron is a major element in the upper continental crust and copper is a trace element [108], and it should therefore be so in the mint dust or clay crucible or cement used. It is thus difficult to explain the higher Cu content relative to Fe in the gold coin studied through this contamination process. Moreover, in a given type of material (e.g., dust, clay), major elements tend to be more homogeneous isotopically than trace elements because they are more likely represent mixed reservoirs. This is attested for iron by geological formation representing dust, i.e., loess, that are rather homogeneous isotopically [109]. Such a local workshop contamination should thus rather display a heterogeneous Cu isotopic signature and a homogeneous Fe isotopic composition, which is exactly the opposite of what is observed in the studied gold coins (**Fig 4**). Hence, it appears that the most likely interpretation of our isotopic results in the dinars studied is that Pb comes from the local, North African workshops contamination and that Cu and Fe isotopes reflect isotopically heavy and heterogeneous gold sources, with a large range of 4‰ for Cu isotopes and more than 2‰ for Fe isotopes, with shorter length-scale heterogeneity for the latter. As raised above, this likely denotes superficial gold ore deposits, known to be more abundant in West African than Southern European gold sources. Moreover, such interpretation of elemental and isotopic data agrees well with the available historical knowledge of the Islamic gold sources.

Two main historical issues were raised with our study. The first is the location of the exploited gold processing. If the gold used has a West African origin, as suggested by the stable isotopes, we must consider that the extracted/collected gold crossed the desert to the North not only to be minted (otherwise, the lead isotopes would have shown signatures compatible with West African gold) but also to be processed. This hypothesis, although logistically challenging as it is much easier to transport processed metal than raw, bulky ore, seems possible given the calculated lead model ages, which are clearly of Mediterranean types. Moreover, except for Egypt, there were no exploitable gold ores North of the Sahara at that time. The second raised issue is the possibility of an older stock of gold reuse, as has been suggested, for earlier Islamic coinage [21]. The level of Cu and Fe isotope heterogeneity found in the Dinars from this study contrasting with the homogeneous and more recent European gold coins contradict this interpretation, however.

## Materials and methods

Performing chemical analyses on gold coins or other ancient artefacts remains challenging due to their rarity and the understandable reluctance of museums to permit destructive analyses. While micro-destructive elemental analyses using LA-ICP-MS are increasingly accepted, isotopic analyses still require more invasive sampling using microdrilling (followed by wet methods, i.e., acid digestion) to ensure high-quality results.

The elemental analyses by LA-ICP-MS were carried out at IRAMAT-CEB (CNRS laboratory, University of Orléans, France) using a sector field, high resolution ICP-MS (Thermo Fisher Scientific, Element XR) coupled with a nanosecond ArF-excimer 193 nm UV laser ablation (Resolution M-50-E, Resonetics) (for usual operating condition: laser energy 6 mJ, ablation rate 6–8 Hz, laser spot diameter 80 µm to keep analytical traces invisible to the naked eye). Coins were ablated in spot mode using the depth profiling mode to overcome potential gold surface enrichment and surface contamination [39,110] (Table S6 in S1 Dataset). Beyond their surface, the coins analyzed for this study appear overall to be homogeneous in elemental composition, given their composition and their method of manufacture (see examples in Blet-Lemarquand and Duyrat [111]). The reproducibility (SD) of the analyses reached at best the order of 3% to 5% relative for Ag and Cu contents in gold, 1–10% for Fe concentrations and 1–20% or sometimes more for Pb concentrations [39,110]. In this study, the standard deviations for Pb concentration determination are random and can be quite high; this is due to the Pb intrinsic nature to segregate in gold (see above) forming micro nodules and to the laser micro-sampling. Accordingly, Pb solubility in gold is extremely low (maximum 0.1 atomic% at 750–900 °C) [112]. To verify Pt and Pd values are not analytical outliers from the LA-ICP-MS measurements, five Islamic coins previously analyzed by Gondonneau [20] and Gondonneau & Guerra [21] using Proton Activation Analysis were remeasured using LA-ICP-MS. The results were consistent (Table S7 in S1 Dataset), as well as with many other coins analyzed with these two methods [93].

In terms of separation chemistry for subsequent isotopic analyses, each coin was sampled at several points using a 1.5 mm diameter drill bit to reach 20 mg and each sample was digested in reverse aqua regia (1/4 HCl + 3/4 HNO_3_). Chromatography was performed with AG1-X4 anionic exchange resin in HCl medium using different molarities to obtain purified fractions of Pb, Cu and Fe. The whole procedure is described in de Palaminy et al.[24] and procedural blanks are the same. Although such a sampling strategy cannot become routine practice for precious heritage objects, significant efforts have been made to address this issue with laser ablation [60].

The isotopic analysis of Fe, Cu and Pb were carried out by MC-ICP-MS (Thermo Fisher Scientific, Neptune Plus) in the GET laboratory (CNRS laboratory, University of Toulouse, France). The accuracy and long-term reproducibility value (2SD) of Fe isotopic measurements was calculated on secondary standard (HEM) and it yields: δ^57^Fe = 0.763 ± 0.060‰ (75 analyses pooled by group of six over 21 months), thus agreeing with previous measurements of this sample [87,113,114]. The long-term reproducibility value (2SD) of Cu isotope measurements was calculated on a secondary standard (pure Cu): δ^65^Cu = 0.228 ± 0.064‰ (39 analyses pooled by group of six over 19 months). For Pb, repeated measurements of the NIST SRM-981 Pb reference material resulted in accuracy of all reported isotopic ratios < 115 ppm (RSD) (200 analyses over 35 months) in agreement with Thirlwall [115] and sample total external uncertainty (that consists of the 2SD on the three triplicates from three different digestions of one sample, the 1878 gold coin) results in ± 0.036 (or 464 ppm RSD) on ^208^Pb/^204^Pb, ± 0.016 (or 497 ppm RSD) on ^207^Pb/^204^Pb, ± 0.019 (or 520 ppm RSD) on ^206^Pb/^204^Pb, ± 0.00041 (or 99 ppm RSD) on ^208^Pb/^206^Pb and ± 0.00006 (or 37 ppm RSD) on ^207^Pb/^206^Pb.

Model ages are calculated for all samples, following the principle outlined by Stacey and Kramers [75], using the method developed by Albarède et al.[71] and incorporating parameters defined by Albarède and Juteau [74]. Model ages are calculated along parameters µ = ^238^U/^204^Pb and κ = ^232^Th/^238^U. The R software [116] was used to construct correlation matrices.

## Supporting information

S1 Dataset All data. Contain Tables S1-S7.
(XLSX)

S1 Supporting Text Correlation matrix on elemental data. Contain S1 Fig, as well as legends for tables from S1 Dataset and references for all SI.
(DOCX)
