# Supplementary material for: New insights into the Trans-Saharan gold trade in the Islamic Middle Ages revealed by multi-isotopic (Pb–Fe–Cu) and elemental characterization of Fatimid gold coins
Source: PLoS One. 2026 Jul 28;21(7):e0353759. doi: 10.1371/journal.pone.0353759 (PMC13411878; doi:10.1371/journal.pone.0353759)
Supplement: S1 Supporting Text — Contain S1 Fig, as well as legends for tables from S1 Dataset and references for all SI. (DOCX) [file pone.0353759.s002.docx]

**Supporting Text S1**

For the manuscript:

**New insights into the Trans-Saharan gold trade in the Islamic Middle Ages revealed by multi-isotopic (Pb–Fe–Cu) and elemental characterization of Fatimid gold coins**

From Louise de Palaminy, Sandrine Baron, Franck Poitrasson, Robert Kool, Maryse Blet-Lemarquand, François-Xavier Fauvelle

This file include:

**Supporting Text S1. Correlation matrix on elemental results.**

**Fig S1. Correlation matrix for the elemental composition of the coins.**

**Legend for Dataset S1**

**Legends for Tables S1-S7 from Dataset S1**

**Supporting Information References**

**Supporting Text S1.** **Correlation matrix on elemental results.**

**Au-Cu-Ag elements.** A clear anticorrelation between Au and both Cu and Ag is highlighted here indicating that higher gold content corresponds to lower silver and copper levels. However, this does not necessarily imply deliberate addition, as Cu and Ag may occur naturally in gold. This result is expected as these three elements are the most abundant.

**Pb-Ag elements.** Lead content can be used to distinguish artificial Au-Ag alloys (with intentional additions of silver) from native gold, which is also a Au-Ag alloy[1]. Native gold, at least that extracted from secondary deposits, is poor in lead (<0.01%), whereas metallic silver, produced from lead-bearing ore or using the cupellation process, almost systematically contains lead, usually between 0.1% and 1%. Here, lead contents are low and show a weak correlation with silver (0.33), suggesting there was no voluntary addition of silver in the alloy.

**Cu-Ag-Fe elements.** Copper and silver are strongly correlated (0.83), and both correlate with Fe (0.72 and 0.71, respectively), indicating they follow similar trends. Two interpretations can be drawn: (i) these elements entered the alloy together via a silver addition containing minor Cu and Fe; (ii) their correlation results from metallurgical processes like cementation, which purify gold from Ag and Cu – and may diminish Fe as well[2,3]. Given the fact siderophile and chalcophile elements do not behave similarly and given the weak Pb-Ag link and the low Pb contents, an intentional silver addition seems unlikely, favouring the second hypotheses.

**Bi, Zn, As, Sb, Sn elements.** These trace elements show consistent positive correlations, though not all exceed 0.70. Experimental work shows these elements are depleted during melting and purification[2,3]. Under reducing conditions, these elements could have been taken up into gold such as Fe, As and Cu[4].

**Fig S1.** Correlation matrix for the elemental composition of the coins. Spearman’s coefficient is used[5] and given the small sample size (n = 11), only strong correlations (> 0.70 or < -0.70) are considered. The closer to 1 (dark blue), the more positively correlated elements are, the closer to -1 (dark red), the more negatively correlated elements are, the closer to 0, the less corelated elements are. Circles stand for a value >0.70 (blue) or <-0.70 (red).

**Legend for Dataset S1**

**Dataset S1. All data.** Contains Tables S1-S7.

This is a separate .xls file.

**Legend for Tables S1-S7 from Dataset S1**

**Table S1. Elemental results of the studied dinars.** Au, Ag and Cu are presented in % and the rest is in µg/g.

**Table S2. Pb isotopic values for all ratios for all samples.** Total external reproducibility consists of the 2SD on the three triplicates from three different digestions of one sample (see methods in de Palaminy et al.[6]).

**Table S3. Model ages T obtained with Albarède et al.[7] calculation, along with mu and kappa factors.**

**Table S4. Isotopic values of δ^65^Cu (‰) against NIST 976 for all dinars.**

**Table S5. Isotopic values of δ^57^Fe (‰) and δ^56^Fe (‰) measured against IRMM-14 for all dinars.**

**Table S6. Surface enrichment of coins visualized with chromatograms.**

**Table S7. Comparison between analyses made by Gondonneau[8] and Gondonneau and Guerra[9] by LA-ICP-MS and PAA (Proton Activation Analysis) and LA-ICP-MS analyses made in 2025 by Maryse Blet-Lemarquand at the laboratory IRAMAT (Orléans) on 5 coins from the French National Library (BnF, Paris). Unit is µg/g but % for major elements Cu, Ag and Au.**

**Supporting Information References**

1. Barrandon JN, Poirier J. Les méthodes d’analyse des monnaies d’or. In: L’Or monnayé I. Purification et altération de Rome à Byzance. Morrison C., Brenot C., Barrondon J.-N., Callu J.-P., Poirier J., Halleux R., Eds. CNRS Editions; 1985. p. 17–38. (Cahiers Ernest Babelon; no. 2).

2. Berger D, Brauns M, Brügmann G, Pernicka E, Lockhoff N. Revealing ancient gold parting with silver and copper isotopes: implications from cementation experiments and for the analysis of gold artefacts. Archaeol Anthropol Sci. 2021;13(9):143. doi:10.1007/s12520-021-01369-2

3. Blet-Lemarquand M, Nieto-Pelletier S, Téreygeol F, Suspène A. Are platinum and palladium relevant tracers for ancient gold coins? Archaeometallurgical and archaometric data to study an antique numismatic problem. In: Montero-Ruiz I, Perea Caveda A, editors. Archaeometallurgy in Europe IV. Madrid: Bibliotheca Praehistorica Hispana; 2017. p. 19–28.

4. Hauptmann A, Klein S. Bronze Age gold in Southern Georgia. ArcheoSciences, revue d’Archéométrie. 2009 Dec 31;(33):75–82. doi:10.4000/archeosciences.2037

5. Schober P, Boer C, Schwarte LA. Correlation Coefficients: Appropriate Use and Interpretation. Anesthesia & Analgesia. 2018 May;126(5):1763. doi:10.1213/ANE.0000000000002864

6. de Palaminy L, Poitrasson F, Baron S, Blet-Lemarquand M, Perrière L. Development of a multi-isotopic (Pb, Fe, Cu) analytical protocol in gold matrices for ancient coins provenance studies. J Anal At Spectrom. 2024 Mar 7;39:1302–21. doi:10.1039/D3JA00312D

7. Albarède F, Desaulty AM, Blichert-Toft J. A geological perspective on the use of Pb isotopes in archaeometry. Archaeometry. 2012 Oct;54(5):853–67. doi:10.1111/j.1475-4754.2011.00653.x

8. Gondonneau A. Développement et application des techniques ICP-MS et LA-ICP-MS à la caractérisation de l’or : circulation monétaire entre Orient et Occident dans l’Antiquité et au Moyen-Age [These de doctorat] [Internet]. Université d’Orléans; 2001 [cited 2020 Oct 26]. Available from: http://www.theses.fr/2001ORLE2070

9. Gondonneau A, Guerra MF. The circulation of precious metals in the Arab Empire: the case of the near and the Middle East. Archaeometry. 2002;44(4):573–99. doi:10.1111/1475-4754.00087
