# Supplementary material for: New insights into the Trans-Saharan gold trade in the Islamic Middle Ages revealed by multi-isotopic (Pb–Fe–Cu) and elemental characterization of Fatimid gold coins
Source: PLoS One. 2026 Jul 28;21(7):e0353759. doi: 10.1371/journal.pone.0353759 (PMC13411878; doi:10.1371/journal.pone.0353759)
Supplement: Attachment — Submitted filename: Response to Reviewers.pdf [file pone.0353759.s004.pdf]

<u>Response to Editor and Reviewers</u>

Dear Luca Bondioli,

Dear Reviewers,

Thank you very much for your in-depth revisions, comments and questions on our manuscript. These have been very helpful.

You will find below **our answers in bold**, within the reviews.

Sincerely yours,

Louise de Palaminy

<u>Editor</u>

Thank you for submitting your manuscript to PLOS ONE. After careful consideration, we feel that it has merit but does not fully meet PLOS ONE's publication criteria as it currently stands. Therefore, we invite you to submit a revised version of the manuscript that addresses the points raised during the review process.

The manuscript by de Palaminy and co-authors provides an interesting contribution to our understanding of the origin of gold in Fatimid coinage.

The manuscript is innovative in some of its implications but is not free from certain issues highlighted by the reviewers. In particular, rev2 asks for some clarifications and precisions that I believe need to be provided by the authors in order to strengthen the contribution.

→ These have been addressed accordingly.

As highlighted by rev1, the graphics and language require revisions to make both more readable and clearer.

→ Figures and English language have been improved.

When submitting your revision, we need you to address these additional requirements.

1. Please ensure that your manuscript meets PLOS ONE's style requirements, including those

for file naming. The PLOS ONE style templates can be found at https://journals.plos.org/plosone/s/file?id=wjVg/PLOSOne_formatting_sample_main_body.pdf and https://journals.plos.org/plosone/s/file?id=ba62/PLOSOne_formatting_sample_title_authors_affiliations.pdf

→ This has been verified.

2. In your manuscript, please provide additional information regarding the specimens used in your study. Ensure that you have reported human remain specimen numbers and complete repository information, including museum name and geographic location.

→ All information on our specimen is already in the manuscript cf. p. 7 l. 194-203: "In 2015, a hoard of 2,668 gold coins of this period, one of the largest hoard of its kind ever discovered in the Eastern Mediterranean (weighing 7.5 kg), was found in a shipwreck in the ancient harbor of Caesarea, Israel (Kool et al., 2018). Now housed in the Coin Department of the Israel Antiquities Authority in Jerusalem, this assemblage offers a rare and valuable case study for gold provenance research." and in Table 1.

If permits were required, please ensure that you have provided details for all permits that were obtained, including the full name of the issuing authority, and add the following statement: 'All necessary permits were obtained for the described study, which complied with all relevant regulations.'

→ The following statement have been added in the manuscript: "All necessary permits were obtained for the described study, which complied with all relevant regulations. We would like to thank Avshalom Karasik (Head of the Laboratory) and Michael Saban (Chief Curator) of the IAA for their trust."

3. Please note that PLOS One has specific guidelines on code sharing for submissions in which author-generated code underpins the findings in the manuscript. In these cases, we expect all author-generated code to be made available without restrictions upon publication of the work. Please review our guidelines at https://journals.plos.org/plosone/s/materials-and-software-sharing#loc-sharing-code and ensure that your code is shared in a way that follows best practice and facilitates reproducibility and reuse.

→ This does not apply to our study.

4. Thank you for stating the following financial disclosure:

"Funding for this study was provided by the CNRS (French National Centre for Scientific Research) via the Mission for Transversal and Interdisciplinary Initiatives (MITI), which awarded an 80 | PRIME PhD grant to TRACES laboratory and the Défi Isotope (Sandrine Baron PI, Toulouse University), and with the assistance of the Transversal Scientific Axis (AST) Isotope of the Midi-Pyrénées Observatory (OMP, Toulouse University)."

Please state what role the funders took in the study. If the funders had no role, please state: "The funders had no role in study design, data collection and analysis, decision to publish, or preparation of the manuscript."

If this statement is not correct you must amend it as needed.

Please include this amended Role of Funder statement in your cover letter; we will change the online submission form on your behalf.

→ **Thank you for changing the information regarding the financial disclosure: "Funding for this study was provided by the CNRS (French National Centre for Scientific Research) via the Mission for Transversal and Interdisciplinary Initiatives (MITI), which awarded an 80 | PRIME PhD grant to TRACES laboratory and the Défi Isotope (Sandrine Baron PI, Toulouse University), and with the assistance of the Transversal Scientific Axis (AST) Isotope of the Midi-Pyrénées Observatory (OMP, Toulouse University). The funders had no role in study design, data collection and analysis, decision to publish, or preparation of the manuscript."**

5. We note that Figure 1 in your submission contain [map/satellite] images which may be copyrighted. All PLOS content is published under the Creative Commons Attribution License (CC BY 4.0), which means that the manuscript, images, and Supporting Information files will be freely available online, and any third party is permitted to access, download, copy, distribute, and use these materials in any way, even commercially, with proper attribution. For these reasons, we cannot publish previously copyrighted maps or satellite images created using proprietary data, such as Google software (Google Maps, Street View, and Earth). For more information, see our copyright guidelines: http://journals.plos.org/plosone/s/licenses-and-copyright.

We require you to either (1) present written permission from the copyright holder to publish these figures specifically under the CC BY 4.0 license, or (2) remove the figures from your submission:

(1) You may seek permission from the original copyright holder of Figure 1 to publish the content specifically under the CC BY 4.0 license.
Please upload the completed Content Permission Form or other proof of granted permissions as an "Other" file with your submission.
In the figure caption of the copyrighted figure, please include the following text: “Reprinted from [ref] under a CC BY license, with permission from [name of publisher], original copyright [original copyright year].”
→ The file content-permission-form_signed.pdf has been uploaded and the following text has been added: “Reprinted from Fauvelle (2018) under a CC BY license, with permission from François-Xavier Fauvelle, original copyright CC BY 2018.”

6. We note that there is identifying data in the Supporting Information file < Supporting Information.zip>. Due to the inclusion of these potentially identifying data, we have removed this file from your file inventory. Prior to sharing human research participant data, authors should consult with an ethics committee to ensure data are shared in accordance with participant consent and all applicable local laws.
Data sharing should never compromise participant privacy. It is therefore not appropriate to publicly share personally identifiable data on human research participants. The following are examples of data that should not be shared:
-Name, initials, physical address
-Ages more specific than whole numbers
-Internet protocol (IP) address
-Specific dates (birth dates, death dates, examination dates, etc.)
-Contact information such as phone number or email address
-Location data
-ID numbers that seem specific (long numbers, include initials, titled “Hospital ID”) rather than random (small numbers in numerical order)
Data that are not directly identifying may also be inappropriate to share, as in combination they can become identifying. For example, data collected from a small group of participants, vulnerable populations, or private groups should not be shared if they involve indirect identifiers (such as sex, ethnicity, location, etc.) that may risk the identification of study participants.
Additional guidance on preparing raw data for publication can be found in our Data Policy (https://journals.plos.org/plosone/s/data-availability#loc-human-research-participant-data-

and-other-sensitive-data) and in the following article:
http://www.bmj.com/content/340/bmj.c181.long.
Please remove or anonymize all personal information, ensure that the data shared are in accordance with participant consent, and re-upload a fully anonymized data set. Please note that spreadsheet columns with personal information must be removed and not hidden as all hidden columns will appear in the published file.
If the reviewer comments include a recommendation to cite specific previously published works, please review and evaluate these publications to determine whether they are relevant and should be cited. There is no requirement to cite these works unless the editor has indicated otherwise.
→ It seems to be a mistake, as no personal identification data has been included in our Supplementary Information file.

Reviewer #1:

The paper presents novel and interesting chemical and isotopic data on gold Fatimid coins, found in the 2015 Caesarea hoard. The adopted multi-analytical approach (minor and trace elements, Pb-Cu-Fe isotopic ratios) correctly addresses the complex issue of gold tracing, which is part of the current discussion on how to interpret the measured geochemical parameters measured on gold in terms of ore provenance, metal stocks, and metallurgical procesess. It was especially fit to read the paper while visiting some of the galamsey sites that are presently a source of Ghanaian gold because of illegal Chinese miners.

Although the archaeological, historical, and geological contexts are rather well delineated, the article is not easy to follow unless the reader is well acquainted with the fine details of gold geology and metallurgy. As a matter of fact the paper possibly raises more questions than answers, since many of the measured parameters and trends can be (and indeed they are) potentially interpreted and discussed in different ways. Therefore, albeit the paper may be hard reading and possibly confusing for many readers, it has the value of showing and discussing in detail the complexity of the interpretation of gold geochemical data, and on this basis I strongly suggest publication. The provided references are also a good review of knowledge on recent

archaeometric gold studies.

The English is good, but it could be smoothed a little. Several adverbial phrases (for instance) and conjunctions (although, either, ...) are misplaced or used inappropriately.

→ **Language has been checked by native speaker.**

Fig. 3 in the version I retrieved is highly compact and almost unreadable. Please make sure the numbers and legends will be readable when printed.

→ **The numbers and legends have been enlarged.**

Reviewer #2:

This manuscript deals with a multi-proxy study of medieval Islamic gold coins with the explicit aim to determine the provenance of the gold. However, it was only possible to identify two distinct metal stocks, differentiated by PGE concentrations and Cu isotopes. However, the stated relationship with West African gold mineralisations based on Cu and Fe isotope ratios is without substance as it is generally agreed that these two isotope system are no indicators of provenance but rather of ore type (e.g. Rose et al 2019: Questioning Fe isotopes as a provenance tool ...JAS 101, Penanes et al 2023: Limitations in using the Cu isotopic composition of minerals from ancient copper mines for archaeometric purposes JAAS 38). Most applications of Cu isotopes in economic geology and archaeology are based on the circumstance that Cu isotopes fractionate due to Cu changing its redox state during weathering of ore deposits and that values of primary ore range roughly between +1.0 to -1.0 regardless of the deposits' locations. These dynamics result in a vertical zonation of the overall Cu isotopic composition within an ore deposit with the amplitude of the fractionation depending on the degree of weathering and how far dissolved Cu is transported within the deposit. Therefore, it is only logical that this behaviour leads to larger intra-mine variability compared to inter-mine variability. The resulting unsuitability of Cu isotopic compositions for the provenance reconstruction of metal artefacts was already stated in several earlier publications The situation is similar or even aggravated for iron isotope ratios. This should be reflected in the text. Furthermore, no attempt was made to analyse native gold samples from the prospective source

regions to check the assumed isotope ratios.

→ We are fully aware of the limitations of Cu and Fe isotopes for strict provenance determination. Both Cu and Fe isotopic compositions primarily reflect redox processes during ore formation and alteration. Our intention is not to use them as standalone provenance tracers, but rather as indicators of ore type. We emphasize the importance of coupling multiple tracers: in our dataset, Cu isotopic signatures appear relatively consistent within individual cities, potentially reflecting a shared supply network. When combined with Pt and Pd concentrations (well-established ore-related indicators for gold) these Cu signatures display interesting correlations, suggesting that they are linked to the ore source. That said, the most positive Cu isotope values are typically associated with oxide and carbonate ores, which correspond to supergene and secondary mineralisations. The exploitation of such near-surface deposits is consistent with medieval written sources describing gold as being relatively easy to "harvest" from the ground. Positive Fe isotope values may be consistent with contributions from banded iron formations (BIF), which are widespread in West Africa. Our interpretation remains cautious: we do not claim that a West African origin must be characterised by high Cu and high Fe isotope values. Rather, we propose that such signatures are compatible with this hypothesis and deserve further investigation. Moreover, the two references cited by the referee are controversial and were criticized in the subsequent literature. We thus added at the end of the introduction: "Even though the stable isotope approach to trace archaeological metals was sometimes criticized on the basis of the finding of large local isotopic heterogeneity of some ore deposits (Rose et al., 2019; Penanes et al., 2023), it appears that, besides possible analytical issues, these previous studies largely ignored the geological and particularly archaeological context of the ore collection and treatment by the ancient miners before metal object production (Milot et al., 2022; Rose et al., 2025),".

→ Regarding the absence of geological gold samples from West Africa in our study, we acknowledge that such analyses would have significantly strengthened the discussion. However, no securely identified ancient gold mines have been documented in the region, making archaeologically relevant comparisons particularly challenging. From an archaeological perspective, direct comparison with modern ores raises chronological concerns, as it introduces a degree of anachronism. Moreover, analysing gold from deep modern hypogene deposits, which were very unlikely to have been exploited in medieval times, would raise its own interpretative limitations. However, many provenance studies

rely on modern geological samples, and a comparison could indeed have been attempted. In our case, practical constraints also played a major role. Access to raw gold material from active West African mining areas is limited: industrial mines are tightly regulated, while shallow artisanal mining is often informal or illegal. In addition, current political instability in several gold-producing regions further complicates access to well-documented geological material. Obtaining geographically and geologically verified gold nuggets is therefore particularly difficult. This study was conceived as exploratory, and it was not possible to address every aspect within its scope. Nevertheless, analysing well-characterised West African gold remains an important objective for future work. Yet, the stable isotope database on Cu and Fe ore is expanding and we use this previous literature for our interpretation throughout the manuscript and notably in the "Interpretation of the Fatimid dinar…" section of the discussion.

It should also be reflected that sample requirements for isotope analysis in ancient gold are prohibitive. The authors used drill samples with a 1.5. mm drill "on several locations". Palaminy et al. [19] state that >20 mg are required. Accordingly, this cannot become a standard method and this should also be explicitly stated.

→ This isotopic study was conducted using wet chemistry protocols, which require macro-destructive sampling. We fully acknowledge that such an approach cannot become routine practice, particularly for heritage objects. We fully acknowledged this in a previous publication in which significant efforts have been made to address this issue: de Palaminy et al. (2025) demonstrated important advances in in situ fs-LA-MC-ICP-MS Cu isotope analyses within gold matrices, and further developments are ongoing. We are confident that less invasive in situ isotopic methods will become increasingly feasible. At the same time, in cases such as coinage which are objects that are often numerous and exceptionally well documented, macro sampling can sometimes be considered within a collaborative framework. For the advancement of knowledge, a carefully reasoned "sacrifice" of a small number of specimens may be acceptable. This tension between heritage preservation and analytical investigation constitutes a genuine and important ethical question, which deserves open discussion between the different actors involved. A sentence has been added on this topic in Materials and method.

Details:

Fig. 2: The ratios of Pd/Au and Pt/Au must be multiplied by a factor of 10exp6.

→ It is indeed common to have a factor on these ratios: we choose a factor (x100) to our modified diagram for a better legibility, which is precised in Fig 2 legend, and added two additional columns with Pt/Au and Pd/Au ratios in Table S1.

Tables S4 and S5: Two decimal figures would be more appropriate. E.g. a statement like 0.289 ± 0.059 does not make sense. It should rather be 0.29 ± 0.06, and so on.

→ Although there is a tradition of giving value with two decimals in archaeometry papers, we disagree because given the precisions obtained, the rounding would unnecessarily degrade our measurements.

Fig. 3: Lead isotope ratios of a single gold mineralisation is taken as representative for whole West Africa?

References: Höhndorf and not Höndorf

→ We added the following statement in the manuscript, in the Fig. 3 legend: "To our knowledge, this is the only Pb isotopic dataset measured directly on gold rather than on host rocks or gold ores reported in the literature, in order to remain consistent with our focus on the metal itself. Such data is very scarce and if this single publication does not fully capture the signature of West African gold, it does give an initial approximation. Furthermore, the ages of mineralisation in the relevant regions are very well constrained by numerous geological studies (see for example Goldfarb et al., 2001; Markwitz, et al., 2016; Masurel et al., 2022)." and the reference for Höhndorf was modified.

Reviewer #3:

The paper is a welcome and valuable contribution towards the clarification of a burning historical problem—the origin of the medieval Islamic gold stock. I am unable to comment on the laboratorial aspect of the work, and I therefore leave this task to other reviewers. I did, nonetheless, find the handling of the data rather candid and unbiased, including the occasional admissions of puzzlement. The conclusions are quite balanced, calling for further study rather than offering definitive answers—which strikes me as very scientifically honest.

As far as the historical framework and interpretation, I have only minor remarks to make, all of which can be easily implemented without altering the structure or impacting the argument of the paper.

On p. 3: the expression “standard Islamic gold currency” seems somewhat anachronistic; nearly nothing was “standard” across the Islamic world between the 7th and 15th centuries. I might suggest: “The dinar, the Arabic term for gold coinage, played a pivotal role…”
Similarly, “remarkably stable” is somewhat misleading—Umayyad and Abbasid dinars were rather uniform at about 4.25 g (though not always of constant fineness), but those of subsequent dynasties somewhat oscillated in weight and fineness (the Almohad dinar was rather heavier, for instance).
→ The reviewer has a point but with an important caveat for our research: major differences in weight of the dinar begin really to occur under the Ayyubid dynasty in the 12/13th centuries and this becomes more pronounced under the Mamluk empire (second half 13th – early 16th cent) when dinars varying greatly in size and weight were essentially small ingots circulating by weight set against a virtual standard mithqal dinar of 4.25 grams. The weight of the Fatimid dinar during the 10th-11th c. is still quite uniform – unpublished results based on the Caesarea hoard's 1728 registered dinars (the largest hoard of Fatimid gold ever discovered in the Mediterranean, certainly from a provenanced find) show that 85% of the dinars fall within the range weight of 3.95 to 4.25 grams and only 2.3% above the weight of 4.25 grams. It is only from the late 12th and later that we see considerable fluctuations in the standardization of the coin's weight: With Saladin (1174 – 1193) and his successors in the 13th century do we see many dinars

appearing with weights above 5 grams and more. This becomes more sustained during the Mamluk period (1260-1517) where dinars are produced weighing as much as 6-7 grams or more. The weight of Mamluk dinars was considerably reduced under the sultan al-Ashraf Barsbay (1422–1438), who introduced dinars of a standard weight of 3.41 grams around 1425/6 based on the weight of the popular Venetian ducats. With regard to standardization of the fineness of the gold in dinars: Researches have shown that also Fatimid dinars contained high standards of gold like the previous Ummayad and Abbasid coinages – 95% gold and higher, which seems to have continued into the Mamluk period.
→ The introduction has been slightly modified taking into account these two remarks, see in the manuscript.

On the same page, change to: "known as Sudan in medieval Arabic sources."
→ It has been changed.

On p. 7: change to "…in Tunisia, and later in Cairo from 969 onwards." (Fustat was the pre-existing Islamic city; the new Fatimid capital and mint were Cairo proper, to Fustat's north-east).
→ Thank you for this precision, it has been modified.
Also, after the Fatimid take-over of Egypt in 969 the Fatimid main mint in Egypt remained located in the ancient Fustat and not in the in the new palace/adminstrative complex built by the new Fatimid rulers north-east of Fustat. There they also had two mints al-Qahirah and al-Qahirah al-Mahrusah but these were minor mints which produced much less coinages. Only later these mints became more important in particular after the fire that destroyed large parts of Fustat in 1164.

On p. 19: "Early Islamic coins minted in North Africa might be initially have been produced by recycling Byzantine and Sassanid gold"—I would strike "Sassanid." Sassanid gold coinage was exceedingly rare and unlikely to have circulated to Roman North Africa; Sassanid silver coinage, on the other hand, was plentiful and mass-recycled in Arab-Sassanid and early Umayyad coinage.
→ Indeed, this is correct: Sassanid gold coinage is extremely rare, and its presence in North Africa is highly improbable. The mistaken reference to Sassanid gold has therefore

been removed, as its remelting cannot reasonably be considered a source for Islamic minting in the region (Schindel et al., 2006 ; Alram et al., 2007)[1].

[1] Schindel N, Sassanian Gold Coinage: An Overview in: A. Gariboldi (ed.), Dal Denarius al Dinar. l'Oriente e la Moneta Romana. Atti dell' Incontro Di Studio, Roma, 16 -18 Settembre 2004, Studi e Materiali 12, Rome 2006: pp. 105-129
Alram, M., Blet-Lemarquand, M., Skjærvø, P.O. (2007). Shapur, King of Kings of Iranians and non-Iranians. In R. Gysylen (Ed.), Res Orientales XVII: Des Indo-Grecs aux Sassanides: Donnees Pour L'histoire et la Geographie Historique. Bures-sur-Yvette: France, pp. 11-40.
