# Supplementary material for: New insights into the Trans-Saharan gold trade in the Islamic Middle Ages revealed by multi-isotopic (Pb–Fe–Cu) and elemental characterization of Fatimid gold coins
Source: PLoS One. 2026 Jul 28;21(7):e0353759. doi: 10.1371/journal.pone.0353759 (PMC13411878; doi:10.1371/journal.pone.0353759)
Supplement: Attachment — Submitted filename: Response to Reviewers_2.docx [file pone.0353759.s005.docx]

**Response to Reviewer n°2**

Dear Reviewer n°2,

Thank you for your comments on our manuscript.
You will find below **our answers in bold**, within the reviews.

Sincerely yours,

Louise de Palaminy

**Reviewer #2:**

Although the text has been substantially improved there remain issues concerning units and uncertainties of the data.

Fig. 2: In the caption it is stated that "Pt and Pd are standardised against Au since these elements are derived from gold. The ratios have been multiplied by a factor of 100 to ensure comparability." It is difficult to comprehend the factor of hundred: Since the concentration of gold is given in percent and those of platinum and palladium in µg/g then the ratios of e.g. palladium are in the range of 0.02 to 0.55 * 10exp-8, if the values of Table S1 are used (!!!). The chart axis must be labeled correctly, regardless of the fact that Gondonneau and Guerra (2002) make the same mistake. This needs to be corrected in Fig. 2 and also in dataset S1_revised to be accepted in a scientific journal.

**🡪 The ratios combine concentrations expressed in different units (wt.% for Au and µg/g for Pt and Pd). Our calculations are correct as for example for coin 566 for Pt: 41(µg/g)/96.4(%)*100=42. The intention was to provide a simple visual normalization, answering the practical question: “If the alloy contained 100 % Au, how many ppm of Pt or Pd would it contain?”.
Fig 2 axes are corrected to Pt/Au*100 and Pd/Au*100 and precisions are made in the caption.**

Furthermore, the use of the concentration unit ppm is discouraged by IUPAC. It is not part of the International System of Units (SI). Why not use mg/kg or µg/g?
**🡪 The unit ppm has been changed to µg/g.**

Copper and iron isotope ratios are mostly given with two decimals, because the uncertainties of measurement are quite large. Here is a recommendation of ASTM International (originally American Society for Testing and Materials):for the use of digits in number formats: The uncertainty of the measurement is expressed by two significant digits if the leading digit in the uncertainty equals 1 or 2, and by one significant digit if the leading digit equals 3 or more. Accordingly, in Table 5 most measurements should be rounded to maximum two decimal places. Interestingly, in Table 4 the copper isotoe ratios are given with three decimals but the uncertainties with two. The copper isotope ratio of one sample (792) as a very large uncertainty and this should alos be corrected along these lines. A value of 1.780 ± 0.128 simply shows that the last digit in the isotope ratio (a zero) is pure speculation. This mus be corected to 1.78 ± 0.13.

🡪 **In the manuscript, numbers of decimals have been modified for 2. In the tables (Excel files in Supp Information), one can actually change the number of digits to their will, so numbers will be left as they are.**
