# Supplementary material for: New insights into the Trans-Saharan gold trade in the Islamic Middle Ages revealed by multi-isotopic (Pb–Fe–Cu) and elemental characterization of Fatimid gold coins
Source: PLoS One. 2026 Jul 28;21(7):e0353759. doi: 10.1371/journal.pone.0353759 (PMC13411878; doi:10.1371/journal.pone.0353759)
Supplement: Peer review history

# Peer review: Decision Letter 0

**DOI:** 10.1371/journal.pone.0353759.r001

**Authors:** Luca Bondioli

23 Feb 2026

PONE-D-26-02097

New insights into the Trans-Saharan gold trade in the Islamic Middle Ages revealed by multi-isotopic (Pb–Fe–Cu) and elemental characterization of Fatimid gold coins

PLOS One

Dear Dr. de Palaminy,

Thank you for submitting your manuscript to PLOS ONE. After careful consideration, we feel that it has merit but does not fully meet PLOS ONE’s publication criteria as it currently stands. Therefore, we invite you to submit a revised version of the manuscript that addresses the points raised during the review process.

The manuscript by de Palaminy and co-authors provides an interesting contribution to our understanding of the origin of gold in Fatimid coinage.

The manuscript is innovative in some of its implications but is not free from certain issues highlighted by the reviewers. In particular, rev2 asks for some clarifications and precisions that I believe need to be provided by the authors in order to strengthen the contribution.

As highlighted by rev1, the graphics and language require revisions to make both more readable and clearer.

Please submit your revised manuscript by Apr 09 2026 11:59PM. If you will need more time than this to complete your revisions, please reply to this message or contact the journal office at plosone@plos.org. When you're ready to submit your revision, log on to https://www.editorialmanager.com/pone/ and select the 'Submissions Needing Revision' folder to locate your manuscript file.

Please include the following items when submitting your revised manuscript:

- A letter that responds to each point raised by the academic editor and reviewer(s). You should upload this letter as a separate file labeled 'Response to Reviewers'.
- A marked-up copy of your manuscript that highlights changes made to the original version. You should upload this as a separate file labeled 'Revised Manuscript with Track Changes'.
- An unmarked version of your revised paper without tracked changes. You should upload this as a separate file labeled 'Manuscript'.

-->If you would like to make changes to your financial disclosure, please include your updated statement in your cover letter. Guidelines for resubmitting your figure files are available below the reviewer comments at the end of this letter.

If applicable, we recommend that you deposit your laboratory protocols in protocols.io to enhance the reproducibility of your results. Protocols.io assigns your protocol its own identifier (DOI) so that it can be cited independently in the future. For instructions see: https://journals.plos.org/plosone/s/submission-guidelines#loc-laboratory-protocols. Additionally, PLOS ONE offers an option for publishing peer-reviewed Lab Protocol articles, which describe protocols hosted on protocols.io. Read more information on sharing protocols at https://plos.org/protocols?utm_medium=editorial-email&utm_source=authorletters&utm_campaign=protocols.

We look forward to receiving your revised manuscript.

Kind regards,

Luca Bondioli, PH.D.

Academic Editor

PLOS One

Journal requirements:

When submitting your revision, we need you to address these additional requirements.

1. Please ensure that your manuscript meets PLOS ONE's style requirements, including those for file naming. The PLOS ONE style templates can be found at

https://journals.plos.org/plosone/s/file?id=wjVg/PLOSOne_formatting_sample_main_body.pdf and

https://journals.plos.org/plosone/s/file?id=ba62/PLOSOne_formatting_sample_title_authors_affiliations.pdf

2. In your manuscript, please provide additional information regarding the specimens used in your study. Ensure that you have reported human remain specimen numbers and complete repository information, including museum name and geographic location.

If permits were required, please ensure that you have provided details for all permits that were obtained, including the full name of the issuing authority, and add the following statement:

'All necessary permits were obtained for the described study, which complied with all relevant regulations.'

If no permits were required, please include the following statement:

'No permits were required for the described study, which complied with all relevant regulations.'

For more information on PLOS One's requirements for paleontology and archeology research, see https://journals.plos.org/plosone/s/submission-guidelines#loc-paleontology-and-archaeology-research.

3. Please note that PLOS One has specific guidelines on code sharing for submissions in which author-generated code underpins the findings in the manuscript. In these cases, we expect all author-generated code to be made available without restrictions upon publication of the work. Please review our guidelines at https://journals.plos.org/plosone/s/materials-and-software-sharing#loc-sharing-code and ensure that your code is shared in a way that follows best practice and facilitates reproducibility and reuse.

4. Thank you for stating the following financial disclosure:

“Funding for this study was provided by the CNRS (French National Centre for Scientific Research) via the Mission for Transversal and Interdisciplinary Initiatives (MITI), which awarded an 80 | PRIME PhD grant to TRACES laboratory and the Défi Isotope (Sandrine Baron PI, Toulouse University), and with the assistance of the Transversal Scientific Axis (AST) Isotope of the Midi-Pyrénées Observatory (OMP, Toulouse University).”

Please state what role the funders took in the study.  If the funders had no role, please state: "The funders had no role in study design, data collection and analysis, decision to publish, or preparation of the manuscript."

If this statement is not correct you must amend it as needed.

Please include this amended Role of Funder statement in your cover letter; we will change the online submission form on your behalf.

5. We note that Figure 1 in your submission contain [map/satellite] images which may be copyrighted. All PLOS content is published under the Creative Commons Attribution License (CC BY 4.0), which means that the manuscript, images, and Supporting Information files will be freely available online, and any third party is permitted to access, download, copy, distribute, and use these materials in any way, even commercially, with proper attribution. For these reasons, we cannot publish previously copyrighted maps or satellite images created using proprietary data, such as Google software (Google Maps, Street View, and Earth). For more information, see our copyright guidelines: http://journals.plos.org/plosone/s/licenses-and-copyright.

We require you to either (1) present written permission from the copyright holder to publish these figures specifically under the CC BY 4.0 license, or (2) remove the figures from your submission:

1. You may seek permission from the original copyright holder of Figure 1 to publish the content specifically under the CC BY 4.0 license.

We recommend that you contact the original copyright holder with the Content Permission Form (http://journals.plos.org/plosone/s/file?id=7c09/content-permission-form.pdf) and the following text:

“I request permission for the open-access journal PLOS ONE to publish XXX under the Creative Commons Attribution License (CCAL) CC BY 4.0 (http://creativecommons.org/licenses/by/4.0/). Please be aware that this license allows unrestricted use and distribution, even commercially, by third parties. Please reply and provide explicit written permission to publish XXX under a CC BY license and complete the attached form.”

Please upload the completed Content Permission Form or other proof of granted permissions as an "Other" file with your submission.

In the figure caption of the copyrighted figure, please include the following text: “Reprinted from [ref] under a CC BY license, with permission from [name of publisher], original copyright [original copyright year].”

2. If you are unable to obtain permission from the original copyright holder to publish these figures under the CC BY 4.0 license or if the copyright holder’s requirements are incompatible with the CC BY 4.0 license, please either i) remove the figure or ii) supply a replacement figure that complies with the CC BY 4.0 license. Please check copyright information on all replacement figures and update the figure caption with source information. If applicable, please specify in the figure caption text when a figure is similar but not identical to the original image and is therefore for illustrative purposes only.

The following resources for replacing copyrighted map figures may be helpful:

USGS National Map Viewer (public domain): http://viewer.nationalmap.gov/viewer/

The Gateway to Astronaut Photography of Earth (public domain): http://eol.jsc.nasa.gov/sseop/clickmap/

Maps at the CIA (public domain): https://www.cia.gov/library/publications/the-world-factbook/index.html and https://www.cia.gov/library/publications/cia-maps-publications/index.html

NASA Earth Observatory (public domain): http://earthobservatory.nasa.gov/

Landsat: http://landsat.visibleearth.nasa.gov/

USGS EROS (Earth Resources Observatory and Science (EROS) Center) (public domain): http://eros.usgs.gov/#

Natural Earth (public domain): http://www.naturalearthdata.com/

6. We note that there is identifying data in the Supporting Information file < Supporting Information.zip>. Due to the inclusion of these potentially identifying data, we have removed this file from your file inventory. Prior to sharing human research participant data, authors should consult with an ethics committee to ensure data are shared in accordance with participant consent and all applicable local laws.

Data sharing should never compromise participant privacy. It is therefore not appropriate to publicly share personally identifiable data on human research participants. The following are examples of data that should not be shared:

-Name, initials, physical address

-Ages more specific than whole numbers

-Internet protocol (IP) address

-Specific dates (birth dates, death dates, examination dates, etc.)

-Contact information such as phone number or email address

-Location data

-ID numbers that seem specific (long numbers, include initials, titled “Hospital ID”) rather than random (small numbers in numerical order)

Data that are not directly identifying may also be inappropriate to share, as in combination they can become identifying. For example, data collected from a small group of participants, vulnerable populations, or private groups should not be shared if they involve indirect identifiers (such as sex, ethnicity, location, etc.) that may risk the identification of study participants.

Additional guidance on preparing raw data for publication can be found in our Data Policy (https://journals.plos.org/plosone/s/data-availability#loc-human-research-participant-data-and-other-sensitive-data) and in the following article: http://www.bmj.com/content/340/bmj.c181.long.

Please remove or anonymize all personal information, ensure that the data shared are in accordance with participant consent, and re-upload a fully anonymized data set. Please note that spreadsheet columns with personal information must be removed and not hidden as all hidden columns will appear in the published file.

If the reviewer comments include a recommendation to cite specific previously published works, please review and evaluate these publications to determine whether they are relevant and should be cited. There is no requirement to cite these works unless the editor has indicated otherwise.

Please review your reference list to ensure that it is complete and correct. If you have cited papers that have been retracted, please include the rationale for doing so in the manuscript text, or remove these references and replace them with relevant current references. Any changes to the reference list should be mentioned in the rebuttal letter that accompanies your revised manuscript. If you need to cite a retracted article, indicate the article’s retracted status in the References list and also include a citation and full reference for the retraction notice.

[Note: HTML markup is below. Please do not edit.]

Reviewers' comments:

Reviewer's Responses to Questions

**Comments to the Author**

1. Is the manuscript technically sound, and do the data support the conclusions?

The manuscript must describe a technically sound piece of scientific research with data that supports the conclusions. Experiments must have been conducted rigorously, with appropriate controls, replication, and sample sizes. The conclusions must be drawn appropriately based on the data presented.

Reviewer #1: Yes

Reviewer #2: Partly

Reviewer #3: Yes

**********

2. Has the statistical analysis been performed appropriately and rigorously?

Reviewer #1: Yes

Reviewer #2: N/A

Reviewer #3: I Don't Know

**********

3. Have the authors made all data underlying the findings in their manuscript fully available?

The PLOS Data policy requires authors to make all data underlying the findings described in their manuscript fully available without restriction, with rare exception (please refer to the Data Availability Statement in the manuscript PDF file). The data should be provided as part of the manuscript or its supporting information, or deposited to a public repository. For example, in addition to summary statistics, the data points behind means, medians and variance measures should be available. If there are restrictions on publicly sharing data—e.g. participant privacy or use of data from a third party—those must be specified.

Reviewer #1: Yes

Reviewer #2: Yes

Reviewer #3: Yes

**********

4. Is the manuscript presented in an intelligible fashion and written in standard English?

PLOS ONE does not copyedit accepted manuscripts, so the language in submitted articles must be clear, correct, and unambiguous. Any typographical or grammatical errors should be corrected at revision, so please note any specific errors here.

Reviewer #1: Yes

Reviewer #2: Yes

Reviewer #3: Yes

**********

5. Review Comments to the Author

Please use the space provided to explain your answers to the questions above. You may also include additional comments for the author, including concerns about dual publication, research ethics, or publication ethics. (Please upload your review as an attachment if it exceeds 20,000 characters)

Reviewer #1: The paper presents novel and interesting chemical and isotopic data on gold Fatimid coins, found in the 2015 Caesarea hoard. The adopted multi-analytical approach (minor and trace elements, Pb-Cu-Fe isotopic ratios) correctly addresses the complex issue of gold tracing, which is part of the current discussion on how to interpret the measured geochemical parameters measured on gold in terms of ore provenance, metal stocks, and metallurgical procesess. It was especially fit to read the paper while visiting some of the galamsey sites that are presently a source of Ghanaian gold because of illegal Chinese miners.

Although the archaeological, historical, and geological contexts are rather well delineated, the article is not easy to follow unless the reader is well acquainted with the fine details of gold geology and metallurgy. As a matter of fact the paper possibly raises more questions than answers, since many of the measured parameters and trends can be (and indeed they are) potentially interpreted and discussed in different ways. Therefore, albeit the paper may be hard reading and possibly confusing for many readers, it has the value of showing and discussing in detail the complexity of the interpretation of gold geochemical data, and on this basis I strongly suggest publication. The provided references are also a good review of knowledge on recent archaeometric gold studies.

The English is good, but it could be smoothed a little. Several adverbial phrases (for instance) and conjunctions (although, either, ...) are misplaced or used inappropriately.

Fig. 3 in the version I retrieved is highly compact and almost unreadable. Please make sure the numbers and legends will be readable when printed.

Reviewer #2: This manuscript deals with a multi-proxy study of medieval Islamic gold coins with the explicit aim to determine the provenance of the gold. However, it was only possible to identify two distinct metal stocks, differentiated by PGE concentrations and Cu isotopes. However, the stated relationship with West African gold mineralisations based on Cu and Fe isotope ratios is without substance as it is generally agreed that these two isotope system are no indicators of provenance but rather of ore type (e.g. Rose et al 2019: Questioning Fe isotopes as a provenance tool ...JAS 101, Penanes et al 2023: Limitations in using the Cu isotopic composition of minerals from ancient copper mines for archaeometric purposes JAAS 38). Most applications of Cu isotopes in economic geology and archaeology are based on the circumstance that Cu isotopes fractionate due to Cu changing its redox state during weathering of ore deposits and that values of primary ore range roughly between +1.0 to -1.0 regardless of the deposits’ locations. These dynamics result in a vertical zonation of the overall Cu isotopic composition within an ore deposit with the amplitude of the fractionation depending on the degree of weathering and how far dissolved Cu is transported within the deposit. Therefore, it is only logical that this behaviour leads to larger intra-mine variability compared to inter-mine variability. The resulting unsuitability of Cu isotopic compositions for the provenance reconstruction of metal artefacts was already stated in several earlier publications The situation is similar or even aggravated for iron isotope ratios. This should be reflected in the text. Furthermore, no attempt was made to analyse native gold samples from the prospective source regions to check the assumed isotope ratios.

It should also be reflected that sample requirements for isotope analysis in ancient gold are prohibitive. The authors used drill samples with a 1.5. mm drill "on several locations". Palaminy et al. [19] state that >20 mg are required. Accordingly, this cannot become a standard method and this should also be explicitly stated.

Details:

Fig. 2: The ratios of Pd/Au and Pt/Au must be multiplied by a factor of 10exp6.

Tables S4 and S5: Two decimal figures would be more appropriate. E.g. a statement like 0.289 ± 0.059 does not make sense. It should rather be 0.29 ± 0.06, and so on.

Fig. 3: Lead isotope ratios of a single gold mineralisation is taken as representative for whole West Africa?

References: Höhndorf and not Höndorf

Reviewer #3: The paper is a welcome and valuable contribution towards the clarification of a burning historical problem—the origin of the medieval Islamic gold stock. I am unable to comment on the laboratorial aspect of the work, and I therefore leave this task to other reviewers. I did, nonetheless, find the handling of the data rather candid and unbiased, including the occasional admissions of puzzlement. The conclusions are quite balanced, calling for further study rather than offering definitive answers—which strikes me as very scientifically honest.

As far as the historical framework and interpretation, I have only minor remarks to make, all of which can be easily implemented without altering the structure or impacting the argument of the paper.

On p. 3: the expression “standard Islamic gold currency” seems somewhat anachronistic; nearly nothing was “standard” across the Islamic world between the 7th and 15th centuries. I might suggest: “The dinar, the Arabic term for gold coinage, played a pivotal role…” Similarly, “remarkably stable” is somewhat misleading—Umayyad and Abbasid dinars were rather uniform at about 4.25 g (though not always of constant fineness), but those of subsequent dynasties somewhat oscillated in weight and fineness (the Almohad dinar was rather heavier, for instance). On the same page, change to: “known as Sudan in medieval Arabic sources.”

On p. 7: change to “…in Tunisia, and later in Cairo from 969 onwards.” (Fustat was the pre-existing Islamic city; the new Fatimid capital and mint were Cairo proper, to Fustat’s north-east).

On p. 19: “Early Islamic coins minted in North Africa might be initially have been produced by recycling Byzantine and Sassanid gold”—I would strike “Sassanid.” Sassanid gold coinage was exceedingly rare and unlikely to have circulated to Roman North Africa; Sassanid silver coinage, on the other hand, was plentiful and mass-recycled in Arab-Sassanid and early Umayyad coinage.

**********

6. PLOS authors have the option to publish the peer review history of their article (what does this mean?). If published, this will include your full peer review and any attached files.

If you choose “no”, your identity will remain anonymous but your review may still be made public.

**Do you want your identity to be public for this peer review?**  For information about this choice, including consent withdrawal, please see our Privacy Policy.

Reviewer #1: No

Reviewer #2: No

Reviewer #3: No

**********

[NOTE: If reviewer comments were submitted as an attachment file, they will be attached to this email and accessible via the submission site. Please log into your account, locate the manuscript record, and check for the action link "View Attachments". If this link does not appear, there are no attachment files.]

To ensure your figures meet our technical requirements, please review our figure guidelines: https://journals.plos.org/plosone/s/figures

You may also use PLOS’s free figure tool, NAAS, to help you prepare publication quality figures: https://journals.plos.org/plosone/s/figures#loc-tools-for-figure-preparation.

NAAS will assess whether your figures meet our technical requirements by comparing each figure against our figure specifications.

# Author comment: Author response to Decision Letter 1

**DOI:** 10.1371/journal.pone.0353759.r002

21 May 2026

Dear editor and reviewers,

You will find all answers in the document entitled "Response to Reviewers.pdf"

On behalf of the co-authors,

Thank you very much,

Louise de Palaminy

Attachment Submitted filename: Response to Reviewers.pdf

# Peer review: Decision Letter 1

**DOI:** 10.1371/journal.pone.0353759.r003

**Authors:** Luca Bondioli

8 Jun 2026

PONE-D-26-02097R1

New insights into the Trans-Saharan gold trade in the Islamic Middle Ages revealed by multi-isotopic (Pb–Fe–Cu) and elemental characterization of Fatimid gold coins

PLOS One

Dear Dr. de Palaminy,

Thank you for submitting your manuscript to PLOS ONE. After careful consideration, we feel that it has merit but does not fully meet PLOS ONE’s publication criteria as it currently stands. Therefore, we invite you to submit a revised version of the manuscript that addresses the points raised during the review process.

The manuscript is almost ready to be published, but Rev. 2 made some interesting but minor  comments that I believe are worth taking into consideration for the benefit of the paper itself. Once these corrections have been made, I’m sure the paper will be published promptly.

Please submit your revised manuscript by Jul 23 2026 11:59PM. If you will need more time than this to complete your revisions, please reply to this message or contact the journal office at plosone@plos.org. When you're ready to submit your revision, log on to https://www.editorialmanager.com/pone/ and select the 'Submissions Needing Revision' folder to locate your manuscript file.

Please include the following items when submitting your revised manuscript:

- A letter that responds to each point raised by the academic editor and reviewer(s). You should upload this letter as a separate file labeled 'Response to Reviewers'.
- A marked-up copy of your manuscript that highlights changes made to the original version. You should upload this as a separate file labeled 'Revised Manuscript with Track Changes'.
- An unmarked version of your revised paper without tracked changes. You should upload this as a separate file labeled 'Manuscript'.

If you would like to make changes to your financial disclosure, please include your updated statement in your cover letter. Guidelines for resubmitting your figure files are available below the reviewer comments at the end of this letter.

If applicable, we recommend that you deposit your laboratory protocols in protocols.io to enhance the reproducibility of your results. Protocols.io assigns your protocol its own identifier (DOI) so that it can be cited independently in the future. For instructions see: https://journals.plos.org/plosone/s/submission-guidelines#loc-laboratory-protocols. Additionally, PLOS ONE offers an option for publishing peer-reviewed Lab Protocol articles, which describe protocols hosted on protocols.io. Read more information on sharing protocols at https://plos.org/protocols?utm_medium=editorial-email&utm_source=authorletters&utm_campaign=protocols.

As the corresponding author, your ORCID iD is verified in the submission system and will appear in the published article. PLOS supports the use of ORCID, and we encourage all coauthors to register for an ORCID iD and use it as well. Please encourage your coauthors to verify their ORCID iD within the submission system before final acceptance, as unverified ORCID iDs will not appear in the published article. *Only*  the individual author can complete the verification step; PLOS staff *cannot*  verify ORCID iDs on behalf of authors.

We look forward to receiving your revised manuscript.

Kind regards,

Luca Bondioli, PH.D.

Academic Editor

PLOS One

Journal Requirements:

1. If the reviewer comments include a recommendation to cite specific previously published works, please review and evaluate these publications to determine whether they are relevant and should be cited. There is no requirement to cite these works unless the editor has indicated otherwise.

2. Please review your reference list to ensure that it is complete and correct. If you have cited papers that have been retracted, please include the rationale for doing so in the manuscript text, or remove these references and replace them with relevant current references. Any changes to the reference list should be mentioned in the rebuttal letter that accompanies your revised manuscript. If you need to cite a retracted article, indicate the article’s retracted status in the References list and also include a citation and full reference for the retraction notice.

[Note: HTML markup is below. Please do not edit.]

Reviewers' comments:

Reviewer's Responses to Questions

**Comments to the Author**

1. If the authors have adequately addressed your comments raised in a previous round of review and you feel that this manuscript is now acceptable for publication, you may indicate that here to bypass the “Comments to the Author” section, enter your conflict of interest statement in the “Confidential to Editor” section, and submit your "Accept" recommendation.

Reviewer #2: All comments have been addressed

Reviewer #3: All comments have been addressed

**********

2. Is the manuscript technically sound, and do the data support the conclusions?

The manuscript must describe a technically sound piece of scientific research with data that supports the conclusions. Experiments must have been conducted rigorously, with appropriate controls, replication, and sample sizes. The conclusions must be drawn appropriately based on the data presented.

Reviewer #2: Yes

Reviewer #3: Yes

**********

3. Has the statistical analysis been performed appropriately and rigorously?

Reviewer #2: N/A

Reviewer #3: I Don't Know

**********

4. Have the authors made all data underlying the findings in their manuscript fully available?

The PLOS Data policy requires authors to make all data underlying the findings described in their manuscript fully available without restriction, with rare exception (please refer to the Data Availability Statement in the manuscript PDF file). The data should be provided as part of the manuscript or its supporting information, or deposited to a public repository. For example, in addition to summary statistics, the data points behind means, medians and variance measures should be available. If there are restrictions on publicly sharing data—e.g. participant privacy or use of data from a third party—those must be specified.

Reviewer #2: Yes

Reviewer #3: Yes

**********

5. Is the manuscript presented in an intelligible fashion and written in standard English?

PLOS ONE does not copyedit accepted manuscripts, so the language in submitted articles must be clear, correct, and unambiguous. Any typographical or grammatical errors should be corrected at revision, so please note any specific errors here.

Reviewer #2: Yes

Reviewer #3: Yes

**********

6. Review Comments to the Author

Please use the space provided to explain your answers to the questions above. You may also include additional comments for the author, including concerns about dual publication, research ethics, or publication ethics. (Please upload your review as an attachment if it exceeds 20,000 characters)

Reviewer #2: Although the text has been substantially improved there remain issues concerning units and uncertainties of the data.

Fig. 2: In the caption it is stated that "Pt and Pd are standardised against Au since these elements are derived from gold. The ratios have been multiplied by a factor of 100 to ensure comparability." It is difficult to comprehend the factor of hundred: Since the concentration of gold is given in percent and those of platinum and palladium in µg/g then the ratios of e.g. palladium are in the range of 0.02 to 0.55 * 10exp-8, if the values of Table S1 are used (!!!). The chart axis must be labeled correctly, regardless of the fact that Gondonneau and Guerra (2002) make the same mistake. This needs to be corrected in Fig. 2 and also in dataset S1_revised to be accepted in a scientific journal.

Furthermore, the use of the concentration unit ppm is discouraged by IUPAC. It is not part of the International System of Units (SI). Why not use mg/kg or µg/g?

Copper and iron isotope ratios are mostly given with two decimals, because the uncertainties of measurement are quite large. Here is a recommendation of ASTM International (originally American Society for Testing and Materials):for the use of digits in number formats: The uncertainty of the measurement is expressed by two significant digits if the leading digit in the uncertainty equals 1 or 2, and by one significant digit if the leading digit equals 3 or more. Accordingly, in Table 5 most measurements should be rounded to maximum two decimal places. Interestingly, in Table 4 the copper isotoe ratios are given with three decimals but the uncertainties with two. The copper isotope ratio of one sample (792) as a very large uncertainty and this should alos be corrected along these lines. A value of 1.780 ± 0.128 simply shows that the last digit in the isotope ratio (a zero) is pure speculation. This mus be corected to 1.78 ± 0.13.

Reviewer #3: All the (minor) comments and suggestions made in the previous round were addressed. The article can be published in its present form.

**********

7. PLOS authors have the option to publish the peer review history of their article (what does this mean?). If published, this will include your full peer review and any attached files.

If you choose “no”, your identity will remain anonymous but your review may still be made public.

**Do you want your identity to be public for this peer review?**  For information about this choice, including consent withdrawal, please see our Privacy Policy.

Reviewer #2: No

Reviewer #3: **Yes:**  Lorenzo M. Bondioli

**********

[NOTE: If reviewer comments were submitted as an attachment file, they will be attached to this email and accessible via the submission site. Please log into your account, locate the manuscript record, and check for the action link "View Attachments". If this link does not appear, there are no attachment files.]

To ensure your figures meet our technical requirements, please review our figure guidelines: https://journals.plos.org/plosone/s/figures

You may also use PLOS’s free figure tool, NAAS, to help you prepare publication quality figures: https://journals.plos.org/plosone/s/figures#loc-tools-for-figure-preparation.

NAAS will assess whether your figures meet our technical requirements by comparing each figure against our figure specifications.

# Author comment: Author response to Decision Letter 2

**DOI:** 10.1371/journal.pone.0353759.r004

28 Jun 2026

Reviewer #2:

Although the text has been substantially improved there remain issues concerning units and uncertainties of the data.

Fig. 2: In the caption it is stated that "Pt and Pd are standardised against Au since these elements are derived from gold. The ratios have been multiplied by a factor of 100 to ensure comparability." It is difficult to comprehend the factor of hundred: Since the concentration of gold is given in percent and those of platinum and palladium in µg/g then the ratios of e.g. palladium are in the range of 0.02 to 0.55 * 10exp-8, if the values of Table S1 are used (!!!). The chart axis must be labeled correctly, regardless of the fact that Gondonneau and Guerra (2002) make the same mistake. This needs to be corrected in Fig. 2 and also in dataset S1_revised to be accepted in a scientific journal.

• The ratios combine concentrations expressed in different units (wt.% for Au and µg/g for Pt and Pd). Our calculations are correct as for example for coin 566 for Pt: 41(µg/g)/96.4(%)*100=42. The intention was to provide a simple visual normalization, answering the practical question: “If the alloy contained 100 % Au, how many ppm of Pt or Pd would it contain?”.

Fig 2 axes are corrected to Pt/Au*100 and Pd/Au*100 and precisions are made in the caption.

Furthermore, the use of the concentration unit ppm is discouraged by IUPAC. It is not part of the International System of Units (SI). Why not use mg/kg or µg/g?

• The unit ppm has been changed to µg/g.

Copper and iron isotope ratios are mostly given with two decimals, because the uncertainties of measurement are quite large. Here is a recommendation of ASTM International (originally American Society for Testing and Materials):for the use of digits in number formats: The uncertainty of the measurement is expressed by two significant digits if the leading digit in the uncertainty equals 1 or 2, and by one significant digit if the leading digit equals 3 or more. Accordingly, in Table 5 most measurements should be rounded to maximum two decimal places. Interestingly, in Table 4 the copper isotoe ratios are given with three decimals but the uncertainties with two. The copper isotope ratio of one sample (792) as a very large uncertainty and this should alos be corrected along these lines. A value of 1.780 ± 0.128 simply shows that the last digit in the isotope ratio (a zero) is pure speculation. This mus be corected to 1.78 ± 0.13.

• In the manuscript, numbers of decimals have been modified for 2. In the tables (Excel files in Supp Information), one can actually change the number of digits to their will, so numbers will be left as they are.

Attachment Submitted filename: Response to Reviewers_2.docx

# Editor report: Decision Letter 2

**DOI:** 10.1371/journal.pone.0353759.r005

**Authors:** Luca Bondioli

29 Jun 2026

New insights into the Trans-Saharan gold trade in the Islamic Middle Ages revealed by multi-isotopic (Pb–Fe–Cu) and elemental characterization of Fatimid gold coins

PONE-D-26-02097R2

Dear Dr. de Palaminy,

We’re pleased to inform you that your manuscript has been judged scientifically suitable for publication and will be formally accepted for publication once it meets all outstanding technical requirements.

Within one week, you’ll receive an e-mail detailing the required amendments. When these have been addressed, you’ll receive a formal acceptance letter and your manuscript will be scheduled for publication.

An invoice will be generated when your article is formally accepted. Please note, if your institution has a publishing partnership with PLOS and your article meets the relevant criteria, all or part of your publication costs will be covered. Please make sure your user information is up-to-date by logging into Editorial Manager at Editorial Manager® and clicking the ‘Update My Information' link at the top of the page. For questions related to billing, please contact billing support.

If your institution or institutions have a press office, please notify them about your upcoming paper to help maximize its impact. If they’ll be preparing press materials, please inform our press team as soon as possible -- no later than 48 hours after receiving the formal acceptance. Your manuscript will remain under strict press embargo until 2 pm Eastern Time on the date of publication. For more information, please contact onepress@plos.org.

Kind regards,

Luca Bondioli, PH.D.

Academic Editor

PLOS One

Additional Editor Comments (optional):

Reviewers' comments:

# Editor report: Acceptance letter

**DOI:** 10.1371/journal.pone.0353759.r006

**Authors:** Luca Bondioli

PONE-D-26-02097R2

PLOS One

Dear Dr. de Palaminy,

I'm pleased to inform you that your manuscript has been deemed suitable for publication in PLOS One. Congratulations! Your manuscript is now being handed over to our production team.

At this stage, our production department will prepare your paper for publication. This includes ensuring the following:

* All references, tables, and figures are properly cited

* All relevant supporting information is included in the manuscript submission,

* There are no issues that prevent the paper from being properly typeset

You will receive further instructions from the production team, including instructions on how to review your proof when it is ready. Please keep in mind that we are working through a large volume of accepted articles, so please give us a few days to review your paper and let you know the next and final steps.

Lastly, if your institution or institutions have a press office, please let them know about your upcoming paper now to help maximize its impact. If they'll be preparing press materials, please inform our press team within the next 48 hours. Your manuscript will remain under strict press embargo until 2 pm Eastern Time on the date of publication. For more information, please contact onepress@plos.org.

You will receive an invoice from PLOS for your publication fee after your manuscript has reached the completed accept phase. If you receive an email requesting payment before acceptance or for any other service, this may be a phishing scheme. Learn how to identify phishing emails and protect your accounts at https://explore.plos.org/phishing.

If we can help with anything else, please email us at customercare@plos.org.

Thank you for submitting your work to PLOS ONE and supporting open access.

Kind regards,

PLOS ONE Editorial Office Staff

on behalf of

Dr. Luca Bondioli

Academic Editor

PLOS One
